# Social defeat stress before pregnancy induces depressive-like behaviours and cognitive deficits in adult male offspring: correlation with neurobiological changes

**DOI:** 10.1186/s12868-018-0463-7

**Published:** 2018-10-16

**Authors:** Sheng Wei, Zifa Li, Meng Ren, Jieqiong Wang, Jie Gao, Yinghui Guo, Kaiyong Xu, Fang Li, Dehao Zhu, Hao Zhang, Rongju Lv, Mingqi Qiao

**Affiliations:** 1 Laboratory of Traditional Chinese Medicine Classical Theory, Ministry of Education, Shandong University of Traditional Chinese Medicine, #4655 University Road, University Science Park, Changqing District, Jinan, 250355 China; 2 Department of Neurosurgery, Qilu Hospital of Shandong University and Brain Science Research Institute, Shandong University, Jinan, 250012 China; 3 Laboratory of Behavioural Brain Analysis, Shandong University of Traditional Chinese Medicine, Jinan, 250355 China; 4 Experimental Center, Shandong University of Traditional Chinese Medicine, Jinan, 250355 China; 5 Fengtai Maternal and Children’s Health Hospital of Beijing, Beijing, 100069 China

**Keywords:** Social defeat stress, Stress before pregnancy, Offspring, Behavioural phenotype, Neurobiochemistry

## Abstract

**Background:**

Epidemiological surveys and studies with animal models have established a relationship between maternal stress and affective disorders in their offspring. However, whether maternal depression before pregnancy influences behaviour and related neurobiological mechanisms in the offspring has not been studied.

**Results:**

A social defeat stress (SDS) maternal rat model was established using the resident-intruder paradigm with female specific pathogen-free Wistar rats and evaluated with behavioural tests. SDS maternal rats showed a significant reduction in sucrose preference and locomotor and exploratory activities after 4 weeks of stress. In the third week of the experiment, a reduction in body weight gain was observed in SDS animals. Sucrose preference, open field, the elevated-plus maze, light–dark box, object recognition, the Morris water maze, and forced swimming tests were performed using the 2-month-old male offspring of the female SDS rats. Offspring subjected to pre-gestational SDS displayed enhanced anxiety-like behaviours, reduced exploratory behaviours, reduced sucrose preference, and atypical despair behaviours. With regard to cognition, the offspring showed significant impairments in the retention phase of the object recognition test, but no effect was observed in the acquisition phase. These animals also showed impairments in recognition memory, as the discrimination index in the Morris water maze test in this group was significantly lower for both 1 h and 24 h memory retention compared to controls. Corticosterone, adrenocorticotropic hormone, and monoamine neurotransmitters levels were determined using enzyme immunoassays or radioimmunoassays in plasma, hypothalamus, left hippocampus, and left prefrontal cortex samples from the offspring of the SDS rats. These markers of hypothalamic–pituitary–adrenal axis responsiveness and the monoaminergic system were significantly altered in pre-gestationally stressed offspring. Brain-derived neurotrophic factor (BDNF), cyclic adenosine monophosphate response element binding protein (CREB), phosphorylated CREB (pCREB), and serotonin transporter (SERT) protein levels were evaluated using western blotting with right hippocampus and right prefrontal cortex samples. Expression levels of BDNF, pCREB, and SERT in the offspring were also altered in the hippocampus and in the prefrontal cortex; however, there was no effect on CREB.

**Conclusion:**

We conclude that SDS before pregnancy might induce depressive-like behaviours, cognitive deficits, and neurobiological alterations in the offspring.

## Background

Epidemiological surveys and animal experiments have established connections between maternal stress and changes in the mood and behaviour of their offspring [1–5]. For example, it has been reported that the risks for anxiety, depression, and addiction disorders, are increased in the children of depressed parents compared to those of non-depressed parents [3], and a considerable number of animal experiments have shown that maternal stress during the prenatal period leads to increased depressive and anxiety-like behaviours in the offspring [6–9]. However, the neurobiological mechanisms through which maternal depression prior to pregnancy has these effects on the offspring have not been well studied.

It has been reported that when female rats are exposed to chronic unpredictable stress (CUS) prior to being pregnant, their male offspring are at increased risk of developing depressive-like behaviours, and it has been suggested that such behaviours are due to altered expression of phosphorylated cyclic adenosine monophosphate response element binding protein (pCREB), brain-derived neurotrophic factor (BDNF), and *N*-methyl-d-aspartate receptor (NMDA-R) subunits in the hippocampus [4, 5]. In most experiments, CUS is implemented through the administration of electric shocks or by physically restraining the animal [4, 5], but such physical stressors are more or less artificial and might be regarded as irrelevant to the situations and stressors that humans and animals encounter in everyday life [10]. In contrast to the paradigm mentioned above, the use of social defeat stress (SDS) as a naturalistic psychosocial stressor might be more suitable for inducing maternal depressive-like behaviours [11]. The resident-intruder paradigm is often used to induce SDS in rodents [12, 13], and in such experiments an adult rodent (the intruder) is placed in the cage of an unfamiliar and aggressive individual (the resident). The animals will instinctively fight, and the intruder will usually lose. These experiments are terminated as soon as the intruder shows signs of submissive behaviour so as to minimize injury while ensuring that the psychosocial components of stress are maximized. In rats, social defeat by an aggressive male is a more natural stressor than the physical stressors mentioned above, and such stress results in a variety of molecular, physiological, and behavioural changes in the intruder animal, and many of these changes exist for a long time.

However, the evidence to support the effects of maternal chronic SDS before pregnancy on offspring is still weak. Therefore, there is much interest in understanding the abnormal behaviours of the offspring and the underlying mechanisms induced by the social stress experienced by the mother before pregnancy. The hypothalamic-pituitary-adrenal (HPA)-axis, is responsible for an individual’s ability to cope with stress, and it does so by regulating the production and release of various hormones [14, 15]. Hyperactivity of the HPA axis has been observed in the majority of patients with depression [16, 17]. Furthermore, it is also well documented that corticosteroids modulate emotional behaviours and cognition in animals and humans in a complex manner [18]. On the other hand, the serotonergic and adrenergic systems play critical roles in modulating the functional neural circuits in brain [19, 20] and have been implicated in hippocampal-dependent memory. It has also been shown that the hippocampus, hypothalamus, and prefrontal cortex are involved in the stress response and are the areas most relevant to depression [21, 22] and that these regions play a primary role in the neuroendocrine control of feeding, emotion, and metabolism in adult life [23]. In addition, monoaminergic signalling pathways mainly act via G-proteins that in turn activate adenylyl cyclase, protein kinase A (PKA), and the transcription factor CREB. pCREB can regulate multiple target genes involved in the pathophysiology of depression [4, 6, 24]. Taken together, the HPA axis, serotonergic and adrenergic systems, and monoaminergic signalling pathways have been investigated in the hippocampus, hypothalamus, and prefrontal cortex to evaluate the mechanisms involved in depression.

In this study, we hypothesized that (1) the SDS experienced by dams before pregnancy causes behavioural abnormalities in their male offspring and (2) the behavioural abnormalities observed in the male offspring are related to abnormalities in the HPA axis, monoaminergic system, and signal transduction pathways. To test these hypotheses, dams were subjected to social defeat on a daily basis for 4 weeks (1 week of social isolation and 3 weeks of defeat stress) and then subjected to a series of behavioural tests. Subsequently, the offspring delivered by the stressed dams were selected for behavioural testing (open-field, sucrose preference, elevated plus maze, light–dark box, forced swim, object recognition, and Morris water maze (MWM) tests). The levels of neurotransmitters in different brain regions; the expression of pCREB, serotonin transporter (SERT), and BDNF; and the correlation between behavioural and molecular changes were examined.

## Methods

### Study design

**Fig. 1:**
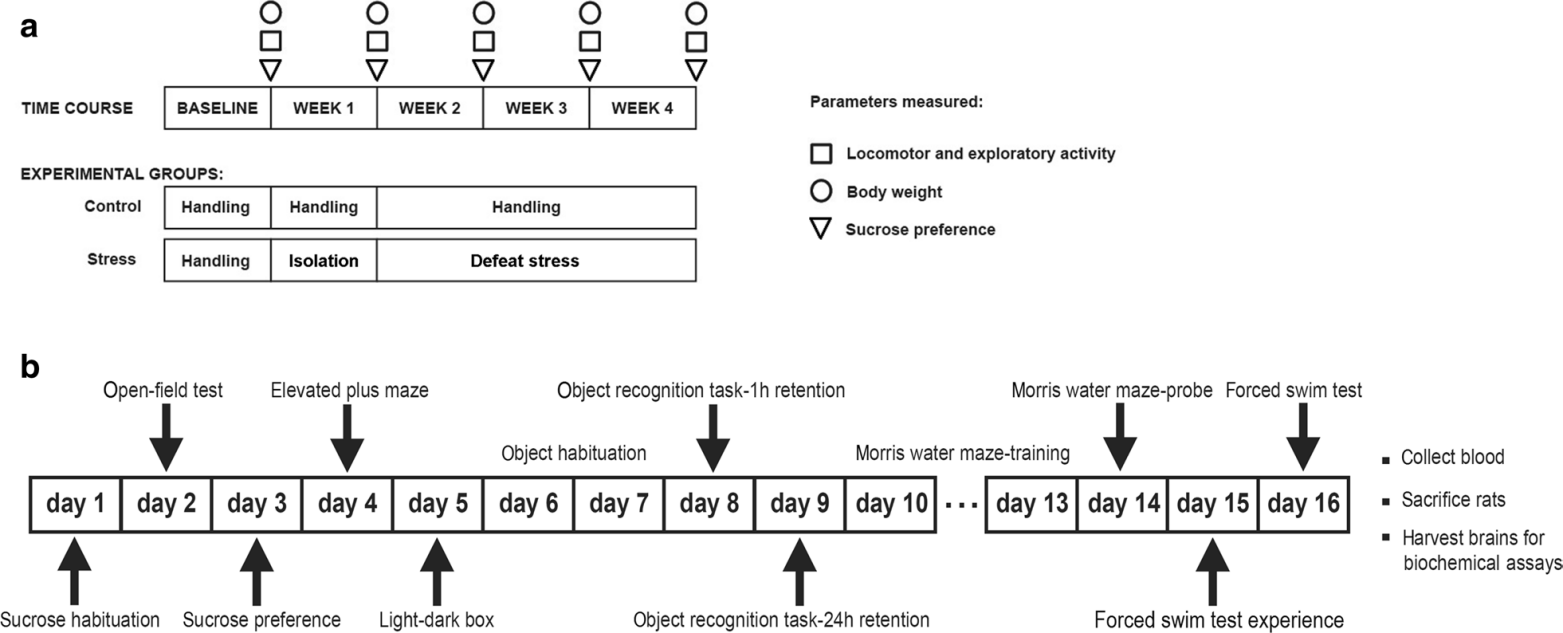
**a** Maternal SDS and behavioural detection protocol. Time points of the open-field test (squares), the body weight measurements (circles), and the sucrose preference test (triangles) are shown. The experimental groups (including control and SDS resident rats) assessed with the three tests are indicated below. **b** Behavioural assessment procedure in the offspring

A maternal SDS rat model was established using the resident-intruder paradigm with female specific pathogen-free Wistar rats and was evaluated by behavioural tests. The maternal SDS procedure and the behavioural analysis process are shown in Fig. 1a. Seven days after the end of the SDS exposure, two female rats were housed with one sexually experienced males of the same strain. These male rats for mating had similar scores on the open-field and sucrose preference tests. The day when sperm were observed in vaginal smears was designated as embryonic day 0. The female rats were then housed separately and allowed to nest and give birth without being disturbed. The day of delivery was designated as postnatal day 0. The pups were removed from their dams at 22 days of age and housed in groups of three or four with males and females kept separate. All experiments described below were performed in the male offspring at 2 months of age. Eight pups from the control and SDS dams were used, with one or two pups used from each dam. The behavioural tests used with the offspring are shown in Fig. 1b. Some methods detailed below (including sucrose preference test, open field test, elevated plus maze, object recognition task, morris water maze test and monoamine neurotransmitter concentration analyses) mainly refer to our previously published work [25].

### Animals and grouping

Twelve female specific pathogen-free (SPF) Wistar rats (resident) weighing 120–150 g and aged 6 weeks, twelve female SPF Wistar rats (intruder) weighing 150–180 g and aged 6 weeks, and six male SPF Wistar rats (used for mating) weighing 180–220 g were provided by Beijing Vital River Experimental Animal Technology Co. Ltd. (SCXK [JING] 2007-0001; Beijing, China).

All animals were housed under a reversed 12/12 h light/dark cycle (lights off at 8:00 a.m. and on at 8:00 p.m.), and food and water were available ad libitum except during the behavioural experiments. The animals were handled daily for 1 week to habituate them to the experimental conditions. The room temperature was maintained at 22 ± 1 °C with 50% humidity. Rats were minimally handled, and soiled bedding was periodically only partially replaced, without removing the rats, so that home-cage odours, nests, etc., were minimally disrupted [26]. All experiments were conducted during the dark phase of the light/dark cycle under dim red light conditions (10:00 a.m. to 5:00 p.m.). Animals were tested using a matched block design with roughly equal numbers of animals in each treatment group in a series of several blocks. The operators were blinded to the experimental design.

Prior to the experiments, all female rats were checked to ensure they had a regular oestrous cycle of 4–5 days and that they showed an equal distribution of the different stages of the oestrous cycle. The oestrous cycle was again examined during the last week of SDS using vaginal smears, as has been previously described [5, 27]. In general, aggressive females with strong bodies and rich fighting experience in non-receptive phases were selected as intruders. Because females in non-receptive phases exhibited aggressive behaviour more than females in other phases [28], these intruders always defeated the resident rats. The intruder rats were housed individually in a separate room under identical conditions as the resident rats. Prior to all stress-inducing procedures, the resident rats were divided into the control group (n = 6) and SDS group (n = 6).

### Weight of female rats

The body weight of each female rat was measured before the start of the SDS (W0) paradigm, and once per week (W1, W2, W3) during the protocol (Fig. 1a). Weight was taken at 9:00 a.m. to 12:00 p.m. every day.

### Sucrose preference test (SPT)

SPTs were performed weekly during the SDS experiment, with the first test being performed at baseline. Two bottles of liquid were provided for the rats to choose from over a 24 h period. One bottle contained a 0.8% sucrose solution and the other contained tap water, and the bottle positions were switched after 12 h. The sucrose preference was determined as the percentage of the total amount of liquid consumed [29]. SPTs were performed in the same manner with the male offspring.

### Open field test (OFT)

The OFT was performed in an open-topped Plexiglas arena (100 cm × 100 cm × 50 cm), Each animal was placed in the centre of the arena and was allowed to explore it for 6 min, and all movements of the animals within the arena were automatically recording using the XR-SuperMaze video tracking and analysis system (Shanghai SOFTMAZE Information Technology Co., Ltd, Shanghai, China). The behaviours within the arena were analysed according to the total distance moved, the distance travelled in the centre and peripheral areas, the time spent in the centre [30, 31], and the number of rearings. The arena was carefully cleaned with 70% ethanol after every test.

### Social defeat stress

The resident-intruder social stress paradigm was used with modifications in which the intruder was the aggressor and the resident was the defeated subject. The social defeat test was conducted for 4 weeks, including 1 week of social isolation (in which dams were fed separately) and 3 weeks of defeat stress (Fig. 1a). The control rats were housed at three rats per cage over the course of the 4 weeks. Experiments were performed using a dark red light (< 2 lx) during the dark period. Female rats in non-receptive phases (metestrus and diestrus) with obvious aggressive behaviour and that were larger in size and heavier in body weight than the residents were selected as intruders [28]. The cage was moved to an observation table, and after approximately 15 min of adaptation a female intruder was transferred from her home cage and introduced into the resident’s cage for a period of 15 min. All fights between intruders and resident rats were observed, and the outcomes of the fights were recorded. The defeat test was conducted once per day for 3 weeks. To avoid individual differences in defeat intensity, residents were confronted each day with a different intruder in a Latin square design.

The defeat sessions were monitored. The quality of the defeat sessions was precisely controlled in order to overcome individual variability within the stress group. ‘Defeat’ was defined as one rat being ‘on top’, or as ‘pinning behaviour’, lasting for approximately 2 s. While the residents and intruders met during the 15 min, they fought many times with different outcomes. Although the intruders were more aggressive and stronger and had more experience in fighting, it was hard to ensure that the residents were defeated every time. Hence, a ‘defeat ratio’ was introduced to make sure that social defeat stress would outweigh individual variability. The defeat ratio was the number of defeats of a resident divided by the total number of fights. Only the residents whose defeat ratio was greater than 80% every day were assigned to the stress group as the pairing dams.

Control animals (n = 6) were handled daily throughout the entire experiment. Handling consisted of picking up each rat, transferring it to the experimental room, and returning it to its home cage.

### Elevated plus maze (EPM) test

The EPM was made of black Perspex with a 10 cm × 10 cm central area and arms 50 cm long and 10 cm wide, and the maze was 50 cm above the floor. The closed arms were enclosed by a 40 cm wall, and the open arms had 0.5 cm edges in order to maximize entries into the open arm [32]. The animals were placed in the centre of the maze facing an open arm [33, 34] and were allowed to explore the maze for 5 min. The time spent in an arm of the maze was recorded starting when two paws had crossed the line into the arm. The number of entries into the arms and the time spent in the arms were used as a measure of the locomotor activity of the rat in the maze.

### Light–dark box (LDB) test

The LDB was made of Plexiglas and consisted of two compartments. The larger section was the bright section (25 cm long × 25 cm wide × 30 cm high) and was illuminated by a 75 W white-light bulb (about 200 lx). The smaller section was the dark section (18 cm long × 25 cm wide × 30 cm high) and was illuminated by a 40 W red-light bulb (about 30 lx). Both bulbs were 45 cm above the floor of the box. The compartments were separated by a wall with a 6.5 cm × 6.5 cm doorway. For each test, the animal was placed in the centre of the bright compartment facing the separating wall. An entry was recorded when the rat moved through the doorway and placed all four paws in the other compartment. The total number of transitions between compartments and the time spent in the illuminated compartment were recorded and used as an indicator of overall activity and anxiety level. The frequencies of grooming, wall climbing, and rearing were also recorded [35].

### Object recognition task (ORT)

Male offspring were tested for four consecutive days in the open-field arena following a previously described ORT protocol [36, 37]. At 10.00 a.m. on days 1 and 2, the animals were habituated to the apparatus by allowing them to freely explore the arena. The open field apparatus was a square box 40 cm wide × 40 cm long × 40 cm high. On day 3, the animals were subjected to a 5 min training session in which they were presented with two identical metal cans that were placed against one wall of the arena. Each rat was released in the middle of the opposite wall with its back to the two cans and allowed to explore the arena and the cans on its own. The time spent exploring each object was recorded using the SuperMaze video tracking system. The time spent exploring the cans was recorded as the time the animal’s nose was within a 2 cm^2^ area surrounding the cans. After the training session, the animals were returned to their home cage for 1 h. The animals were then returned to the arena, only now it contained two different objects. One object was identical to the metal cans used in the training session but that had not been previously used, while the other was a novel metal, glass, or hard plastic object. The time spent exploring each object was recorded over a period of 5 min. On day 4, the rats were tested again for 5 min with the familiar object and a different novel object from that used on day 3. The novel objects were randomized and counterbalanced among all of the tested animals. All objects employed in this experiment were used only once in order to eliminate olfactory cues. Familiar objects were always of the same material, colour, size, and shape, and the unfamiliar objects were always different between day 3 and day 4. The objects and the arena were thoroughly cleaned with 70% ethanol at the end of each experimental session.

The recognition index (RI) is calculated as the time spent investigating the novel object divided by the total time spent exploring the novel and familiar objects [RI = TN/(TN + TF)] and is a measure of novel object recognition and is the main index used for analysing memory (or the response to novelty). An RI greater than 50% indicates more time spent exploring the novel object, while an RI less than 50% indicates more time spent exploring the familiar object. An RI of 50% indicates a null preference.

### Morris water maze (MWM) test

The MWM consisted of a light-blue swimming pool 160 cm in diameter with 70 cm walls filled with tap water to a depth of 50 cm. The water temperature was maintained at 23 ± 2 °C. The SuperMaze system was used to divide the pool into four quadrants (North-West [NW], North-East [NE], South-West [SW], and South-East [SE]) of equal size. A removable square escape platform (10 cm × 10 cm) could be positioned in the quadrants, and the centre of the platform was 30 cm away from the wall and 1.5 cm below the surface of the water such that it was not visible to the swimming rat. The pool was placed in an experiment room that had several external visual cues such as bookshelves and posters, and the pool was kept in the same position throughout the entire experimental period. The SuperMaze video system was used to record the animal’s movements in the pool, including measures of the time until finding the escape platform, the total path length of swimming, and the time spent in each quadrant.

The animals were subjected to a training protocol as described by Plescia et al. [36]. Place learning was conducted over 4 days by training the rats to escape from the water by reaching the hidden platform placed in the SE zone where it was maintained throughout the experimental session. Rats were placed in the pool facing the walls of each quadrant in the following order: SW, NW, NE, and SE. Each animal underwent four trials per day over four consecutive days, and they were allowed to swim until the escape platform was found (escape latency) for a maximum of 120 s. When the platform was reached, they were allowed to rest on it for 15 s. If the animal did not find the escape platform within 120 s, the experimenter guided it gently to the platform where it was allowed to rest for 15 s in order to reinforce the information from the visuo-spatial cues in the environment of the experiment room. The animals were returned to their cages and briefly warmed under a heating lamp during the 5 min interval between trials. The video tracking software recorded the escape latency (s) as a measure of the acquisition and retrieval of the spatial information necessary to reach the platform location, and the path length (m) was recorded as an additional element in the search strategies.

The day after the animal had completed the 4-day place-learning task, it was placed in the pool but without the escape platform. The time spent in each quadrant was recorded (transfer test) to determine the degree of learning in the animals with respect to where the escape platform had been located during the place-learning task.

### Forced swimming test (FST)

The FST was performed as described previously [38]. The animals were individually placed into glass cylinders 18 cm in diameter and 40 cm tall containing 18 cm of water at 23 °C. After a pre-test of 15 min swimming, the rats were transferred to a 30 °C drying environment for 30 min before being returned to their cages. The animals were then returned to the cylinder 24 h later for a 5 min test that was recorded with a video camera. Fresh water was used for each rat, and the cylinder was cleaned after each use. All experiments were performed between 12:00 p.m. and 4:00 p.m, and the videotapes were reviewed by an experimenter who was blinded to the group allocation of the animals. Immobility time was measured, which was defined as when the animal was floating and only moving enough to keep its nostrils above the surface of the water [29].

### Tissue and blood collection

Rats were taken from their home cages on the next day after FST in order to collect tissue and blood samples, and they were sacrificed via decapitation between 8:00 a.m. and 10:00 a.m. Trunk blood was collected into ethylenediaminetetraacetic acid (EDTA) tubes, centrifuged at 1250×*g* (15 min, 4 °C), and plasma was collected and frozen until ACTH and corticosterone levels were determined. The brains were removed from the skull and placed on ice. Using the bregma as a reference landmark, the hippocampus, hypothalamus, and prefrontal cortex were dissected out. The hypothalamus, left hippocampus, and left prefrontal cortex were used for the detection of monoamine neurotransmitters via radioimmunoassays, and the right hippocampus and right prefrontal cortex were used to examine BDNF, CREB, pCREB, and serotonin transporter (SERT) protein levels using western blotting.

### Plasma corticosterone and ACTH analyses

Corticosterone levels were determined using 30 μl of plasma with a commercially available enzyme immunoassay kit (CUSABIO^®^, China), and ACTH levels were assessed using 200 μl of plasma with the HS-ACTH radioimmunoassay kit (CUSABIO^®^, China) as previously described [33]. The inter- and intra-assay coefficients of variation (CVs) for ACTH level determination were both 15%, and the inter- and intra-assay CVs for corticosterone determination were 8% and 10%, respectively.

### Monoamine neurotransmitter concentration analyses

Norepinephrine (NE), serotonin (5-HT), and dopamine (DA) levels in the hypothalamus, hippocampus, and prefrontal cortex were measured in brain tissue samples of recommended volume using commercially available enzyme immunoassay kits (CUSABIO^®^, China) specific for each compound as previously described [33]. The inter- and intra-assay CVs for neurotransmitter concentration measurements were 8% and 10% for NE, 15% and 15% for 5-HT, and 8% and 10% for DA, respectively.

### Western blotting

Tissue samples from the hippocampus and prefrontal cortex of offspring were homogenized in extraction buffer (C500006, Sangon Biotech, Shanghai, China) according to the manufacturer’s instructions. Each sample was adjusted to a final protein concentration of 1 μg/μl, mixed with Laemmeli’s sample buffer, and boiled for 5 min. Samples (40 mg) were loaded onto 8% bisacrylamide gels and separated by sodium dodecyl sulphate polyacrylamide gel electrophoresis (SDS-PAGE). Proteins were electrophoretically transferred from gels to polyvinylidene fluoride (PVDF) membranes that were then incubated with the following primary antibodies: anti-BDNF (1:200 dilution, AV41970, Sigma-Aldrich, St. Louis, MO, USA), anti-SERT (1:200, AG1204, Abgent, San Diego, CA, USA), anti-CREB (1:500 dilution, ab31387, Abcam, Cambridge, MA, USA), anti-pCREB (1:500 dilution, ab32096, Abcam), and anti-GAPDH (1:2500 dilution, ab9485, Abcam). Dilutions of peroxidase-conjugated goat anti-rabbit IgG secondary antibody (1:2000 dilution, sc-2004, Santa Cruz Biotechnology, Dallas, TX, USA) were prepared following the manufacturer’s instructions. Immuno-positive bands were visualized using a chemiluminescent method (G:BOX chemiXR5, SYNGEN, Sacramento, CA, USA), and the band densities were determined with the Gel-Pro32 software [39]. All western blotting experiments were repeated at least three times.

### Statistical analysis

Data were analysed using GraphPad Prism version 7.0.4 (GraphPad Software, Inc., San Diego, California, USA). Outliers were defined as two or more standard deviations from the mean, and these were removed from the analysis [26]. The data were tested for normality (Kolmogorov–Smirnov test) and homoscedasticity (Levene’s test) before being analysed using either unpaired *t*-tests or parametric repeated measures analysis of variance (ANOVA). The results from the behavioural tests were analysed using unpaired *t*-tests or two-way ANOVA, and neurochemical and biochemical data were analysed using unpaired *t*-tests. For all analyses, Bonferroni post hoc tests were performed following ANOVA where appropriate. Data are presented as mean ± standard error of the mean (SEM), and the level of significance for differences determined via ANOVA and post hoc testing was set at p < 0.05.

## Results

### Effects of SDS on body weight gain and behaviour of dams

**Fig. 2:**
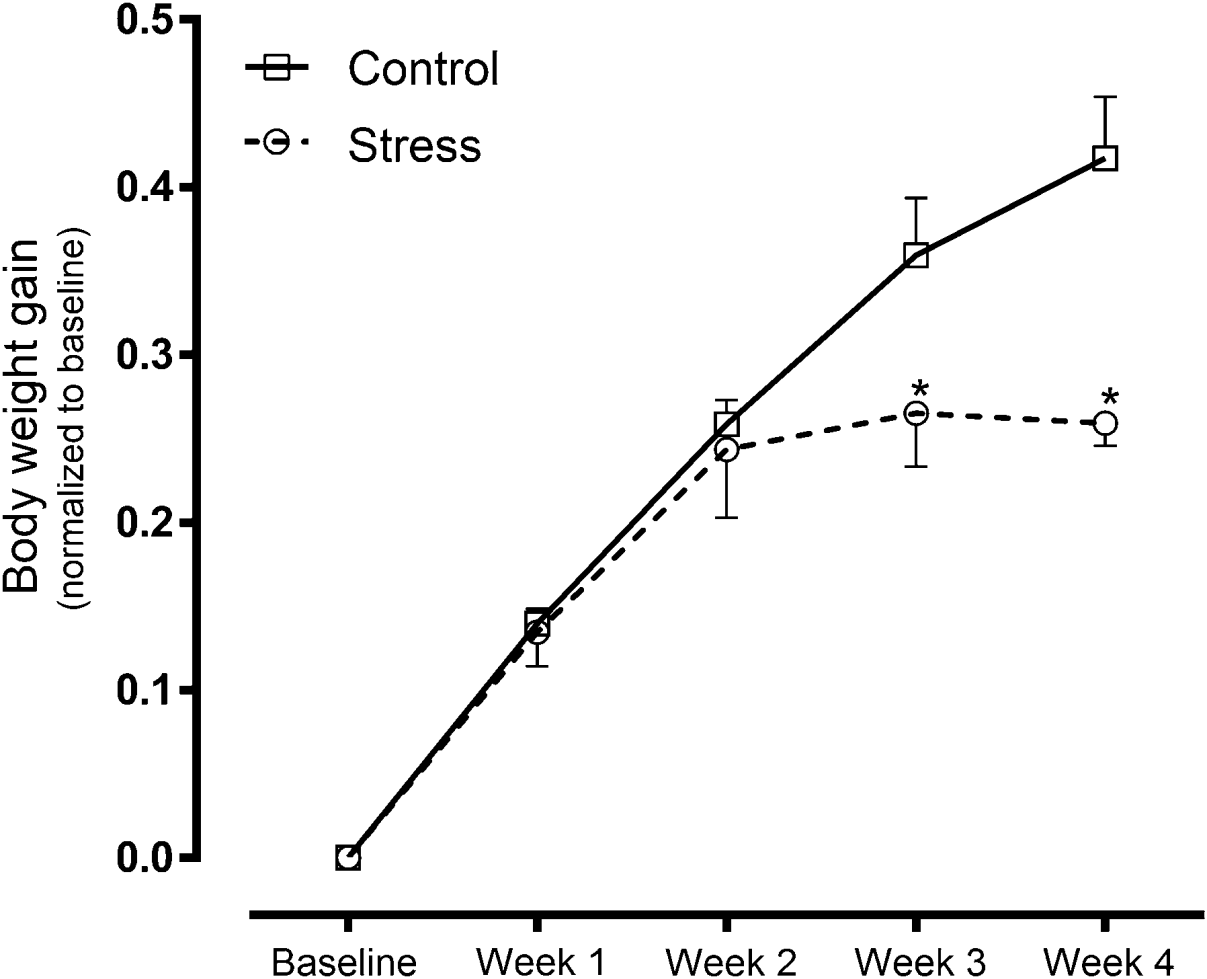
Body weight gain of maternal rats exposed to SDS as well as control rats at 4 weeks after induced stress. Control: control group (hollow square, n = 6); stress: SDS dams (hollow circle, n = 5, outlier number = 1); *p < 0.05

The body weight gain of dams exposed to SDS was found to be significantly reduced compared to controls after 4 weeks of stress (Fig. 2). Statistical analyses revealed a significant effect of stress [F(1, 9) = 5.516, p = 0.0434] and a significant stress × time interaction [F(4, 36) = 5.689, p = 0.0012]. Subsequent Bonferroni post hoc tests confirmed a significant reduction in body weight gain in SDS animals after 3 weeks [t(45) = 2.703, p = 0.0483] or 4 weeks [t(45) = 4.533, p = 0.0002] of experimentation compared to controls.

**Fig. 3:**
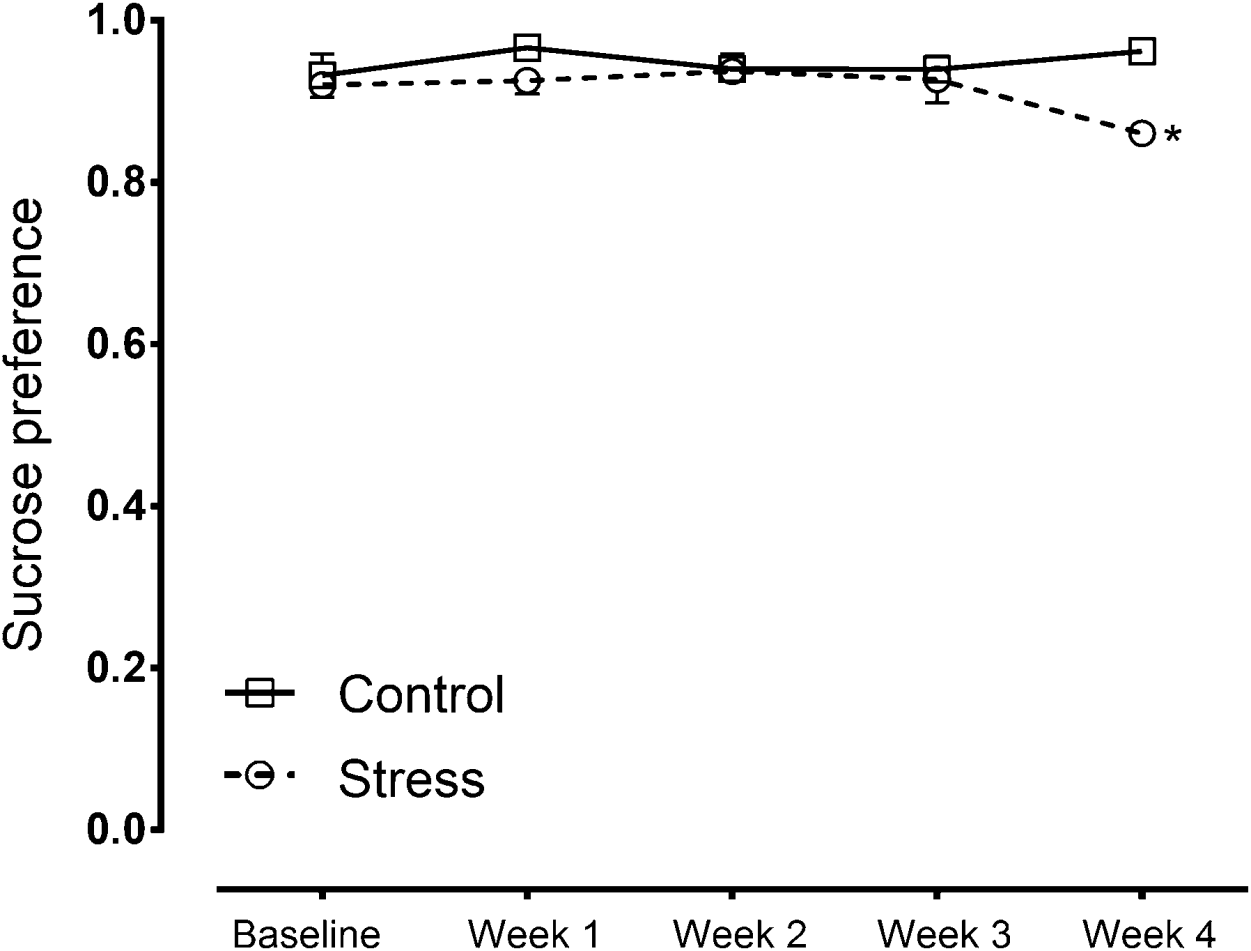
Sucrose preference in maternal rats exposed to SDS as well as control rats at 4 weeks after induced stress. Control: control group (hollow square, n = 6); stress: SDS dams (hollow circle, n = 6); *p < 0.05

At baseline and after 3 weeks of stress, both the SDS and control animals showed similar preferences for sucrose (Fig. 3). However, 4 weeks of stress reduced this preference in SDS animals (Fig. 3), and subsequent Bonferroni post hoc tests showed that the consumption of the 0.8% sucrose solution was significantly lower in the SDS group [t(50) = 4.133, p = 0.0007] than in control rats (Fig. 3). Two-way ANOVA revealed a significant effect of stress [F(1, 10) = 7.04, p = 0.0242].

**Fig. 4:**
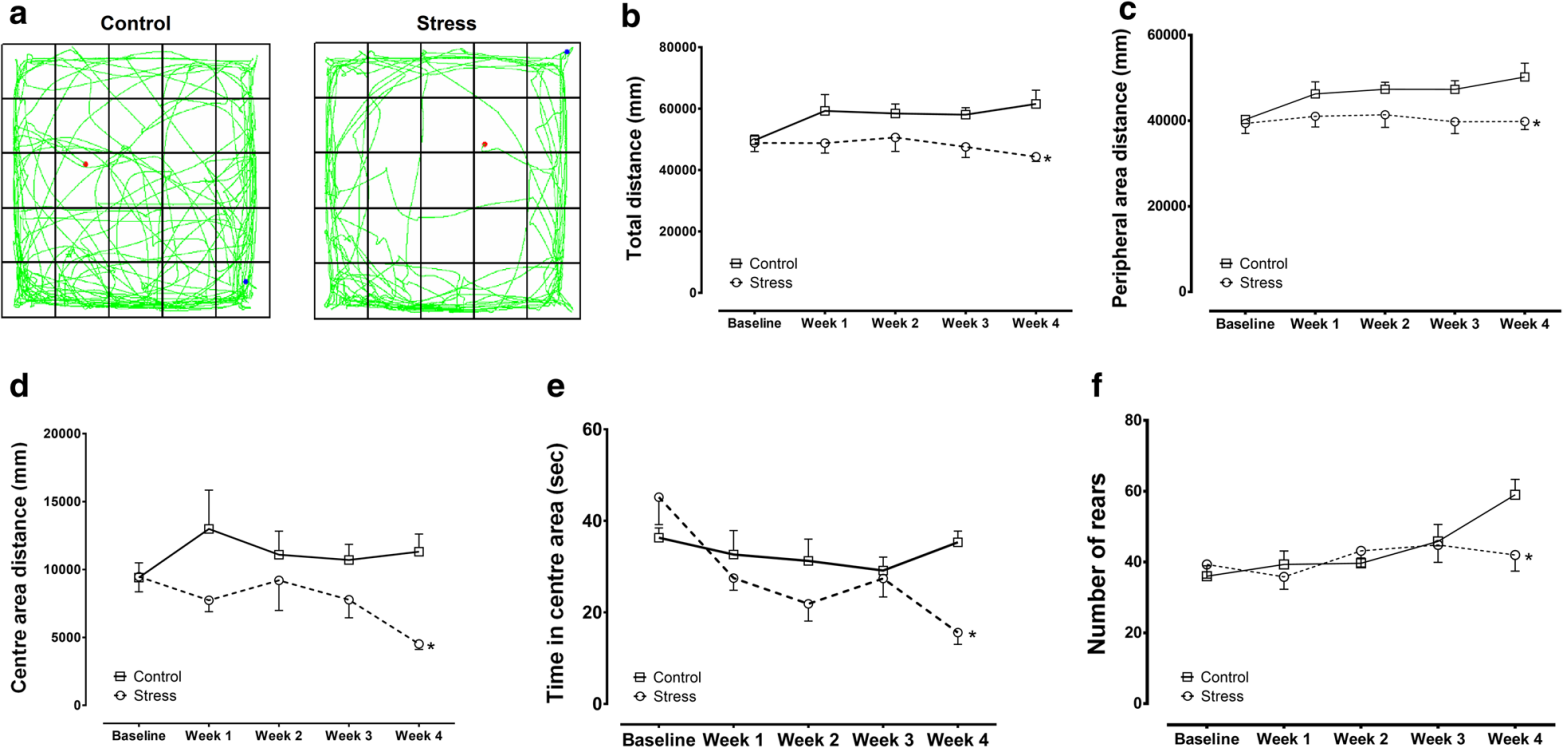
Open-field test results from maternal rats exposed to SDS as well as control rats at 4 weeks after induced stress. **a** Trajectory for both groups. **b** Total distance (mm) travelled in a 6 min period. **c** Peripheral area distance (mm) travelled in a 6 min period. **d** Centre-area distance (mm) travelled in a 6 min period. **e** Time spent (s) in the centre area. **f** Number of rearings. Control: control group (hollow square, n = 6); Stress: SDS dams (hollow circle, n = 6); *p < 0.05

In the OFT, there was a significant difference in the total distance and in the distance travelled in peripheral areas between the control and SDS groups after 4 weeks of stress. Subsequent Bonferroni post hoc tests revealed that the SDS group showed a reduction in the total distance moved and in the distance travelled in the peripheral areas compared to controls (Fig. 4a–c). Two-way ANOVA revealed a significant effect of stress [total distance: F(1, 10) = 19.3, p = 0.0014; Peripheral area distance: F(1, 10) = 19.87, p = 0.0012]. There were also significant differences in the distance travelled in the centre area, in the time spent in the centre area, and in the number of rearings between the groups after 4 weeks of stress, and Bonferroni post hoc tests showed a significant decrease in those measures in the SDS group compared to controls [distance travelled: t(50) = 3.086, p = 0.0165; Time in centre area: t(50) = 3.608, p = 0.0036; Rearings: t(50) = 3.002, p = 0.0209] (Fig. 4a, d–f). Two-way ANOVA revealed a significant effect of stress [F(1, 10) = 10.29, p = 0.0094] on the distance travelled in the centre area, a significant effect of stress × time interaction [F(4, 40) = 3.852, p = 0.0097] and time [F(4, 40) = 5.299, p = 0.0016] on the time spent in the centre area, and a significant effect of time [F(4, 40) = 3.577, p = 0.0138] on the number of rearings.

### Behavioural characterization of offspring subjected to pre-gestational SDS

**Fig. 5:**
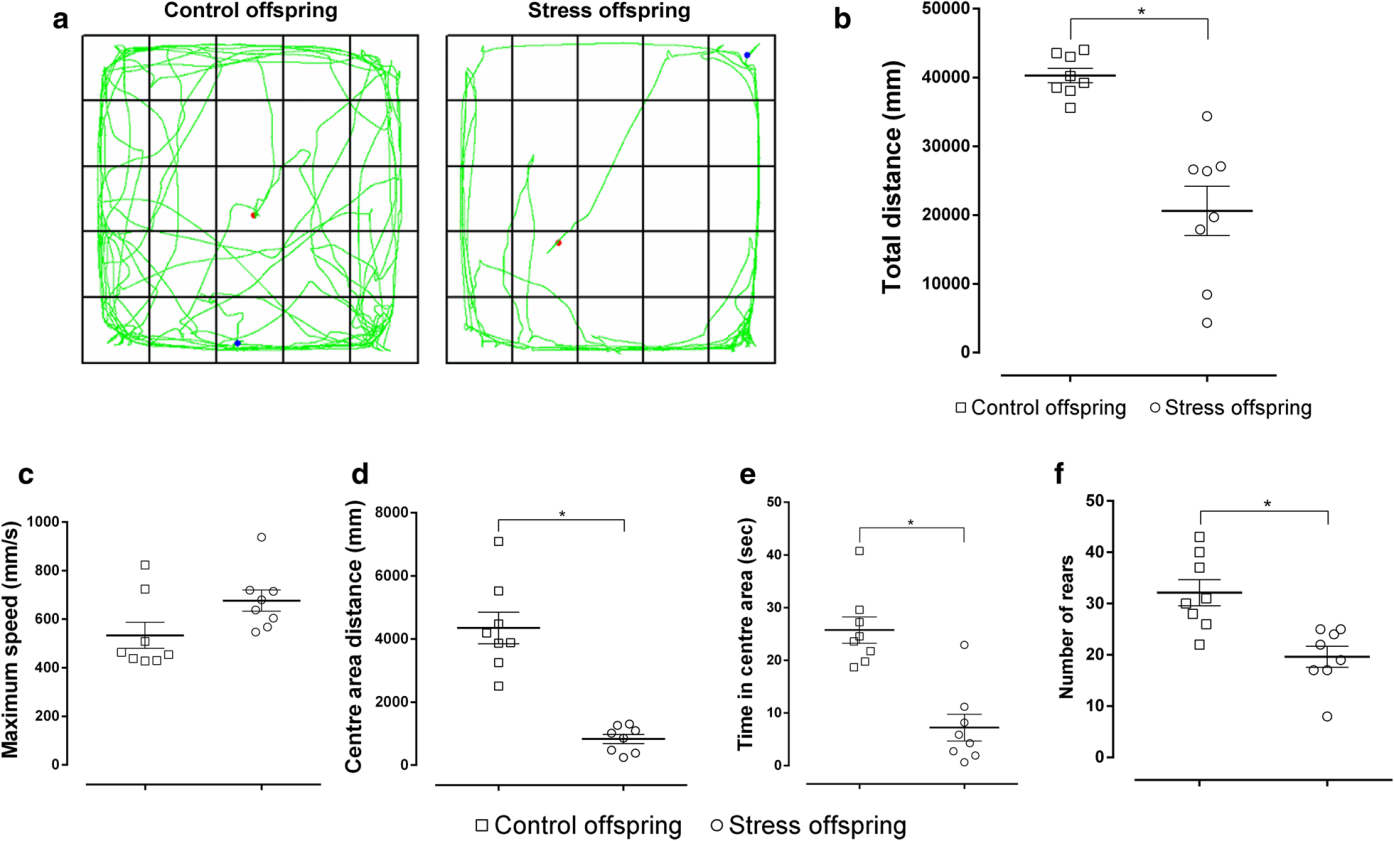
Open-field test results for stress and control offspring. **a** Trajectory for both groups. **b** Total distance (mm) travelled in a 6 min period. **c** Maximum speed (mm/s). **d** Distance travelled (mm) in the centre area in a 6 min period. **e** Time spent (s) in the centre area. **f** Number of rearings. Control offspring: offspring of control group (hollow square, n = 8); Stress offspring: offspring of maternal rats subject to SDS stimulation (hollow circle, n = 8); *p < 0.05

In the OFT, locomotor activity was modified by pre-gestational SDS (Student’s *t*-test). The total path length travelled was 40,277 ± 1062 mm for the control offspring and 20,607 ± 3590 mm for the stressed offspring (Fig. 5a, b). There was no significant difference in the maximum speed between the groups (t(14) = 2.054, p = 0.0591 [Student’s *t*-test], SDS offspring: 533.6 ± 53.95 mm/s vs. control offspring: 676.2 ± 43.68 mm/s; Fig. 5c). The offspring subjected to pre-gestational SDS travelled a reduced distance in the centre area and spent less time in the centre area than the control offspring (distance travelled: t(14) = 6.754, p < 0.05, SDS offspring = 4349 ± 500.3 mm vs. control offspring = 829.4 ± 145.4 mm; Time in centre area: t(14) = 5.185, p < 0.05 [Student’s *t*-test], SDS offspring = 25.74 ± 2.499 s vs. control offspring = 7.221 ± 2.553 s; Fig. 5a, d, e). The pre-gestationally stressed offspring also reared less than the controls (t(14) = 3.823, p = 0.0019 [Student’s *t*-test], SDS offspring = 32.13 ± 2.560 rearings vs. control offspring = 19.63 ± 2.035 rearings; Fig. 5a, f).

**Fig. 6:**
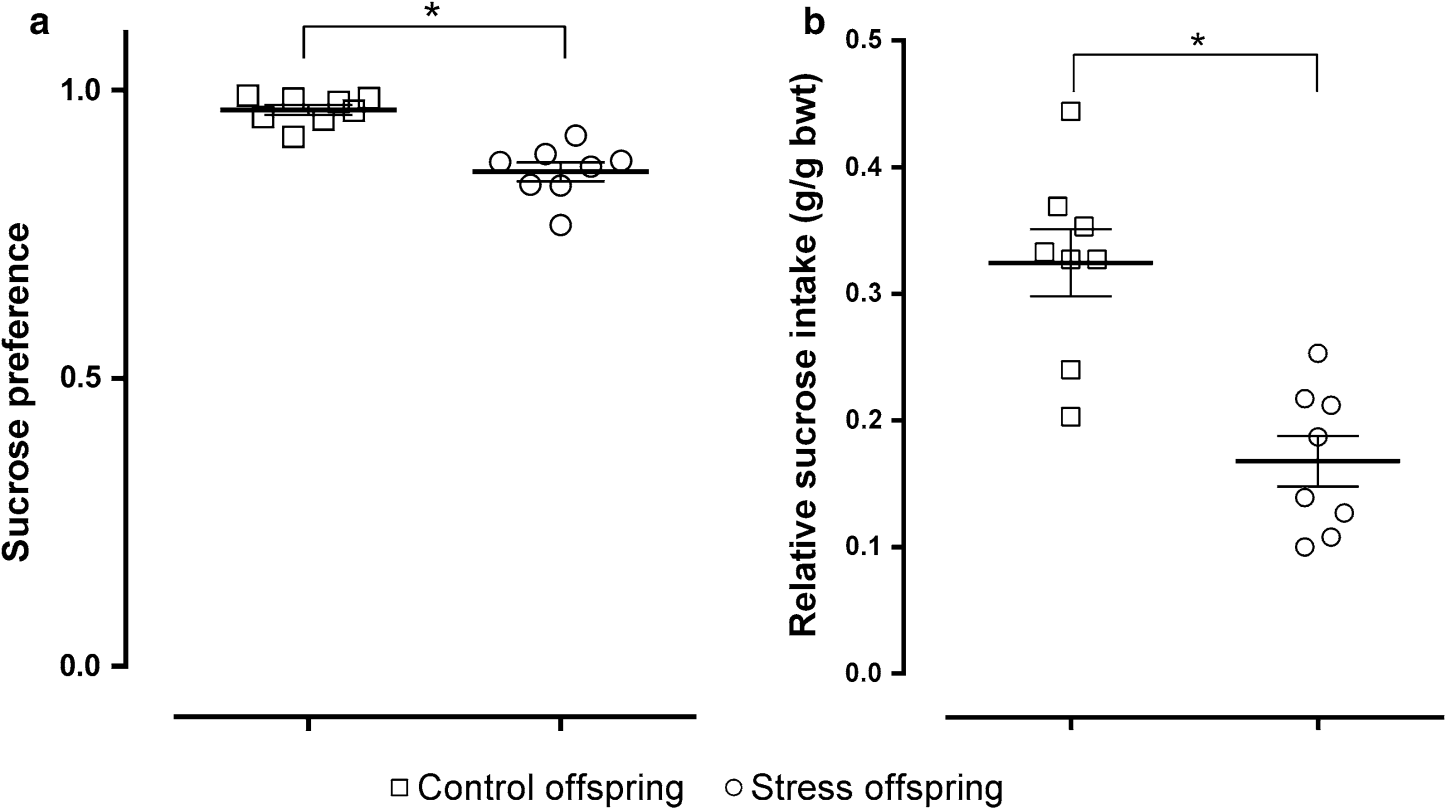
Sucrose preference results for stress and control offspring. **a** Preference for the sucrose solution [sucrose intake (g)/total liquid intake (g)]. **b** Relative preference for the sucrose solution [sucrose intake (g)/body weight (g)]. Control offspring: offspring of control group (hollow square, n = 8); stress offspring: offspring of maternal rats subject to SDS stimulation (hollow circle, n = 8); bwt: body weight; *p < 0.05

In the SPT, although all animals tested showed preference for the sucrose solution compared to water, sucrose intake (sucrose preference index and relative sucrose intake) was reduced in the offspring of SDS dams compared with controls (sucrose preference index: t(14) = 5.782, p < 0.05, SDS offspring = 0.9656 ± 0.008635 g/g bwt [body weight] vs. control offspring = 0.8581 ± 0.01647 g/g bwt; relative sucrose intake: t(14) = 4.717, p = 0.0003 [Student’s *t*-test], SDS offspring = 0.3245 ± 0.02641 g/g bwt vs. control offspring = 0.1679 ± 0.02012 g/g bwt; Fig. 6a, b).

**Fig. 7:**
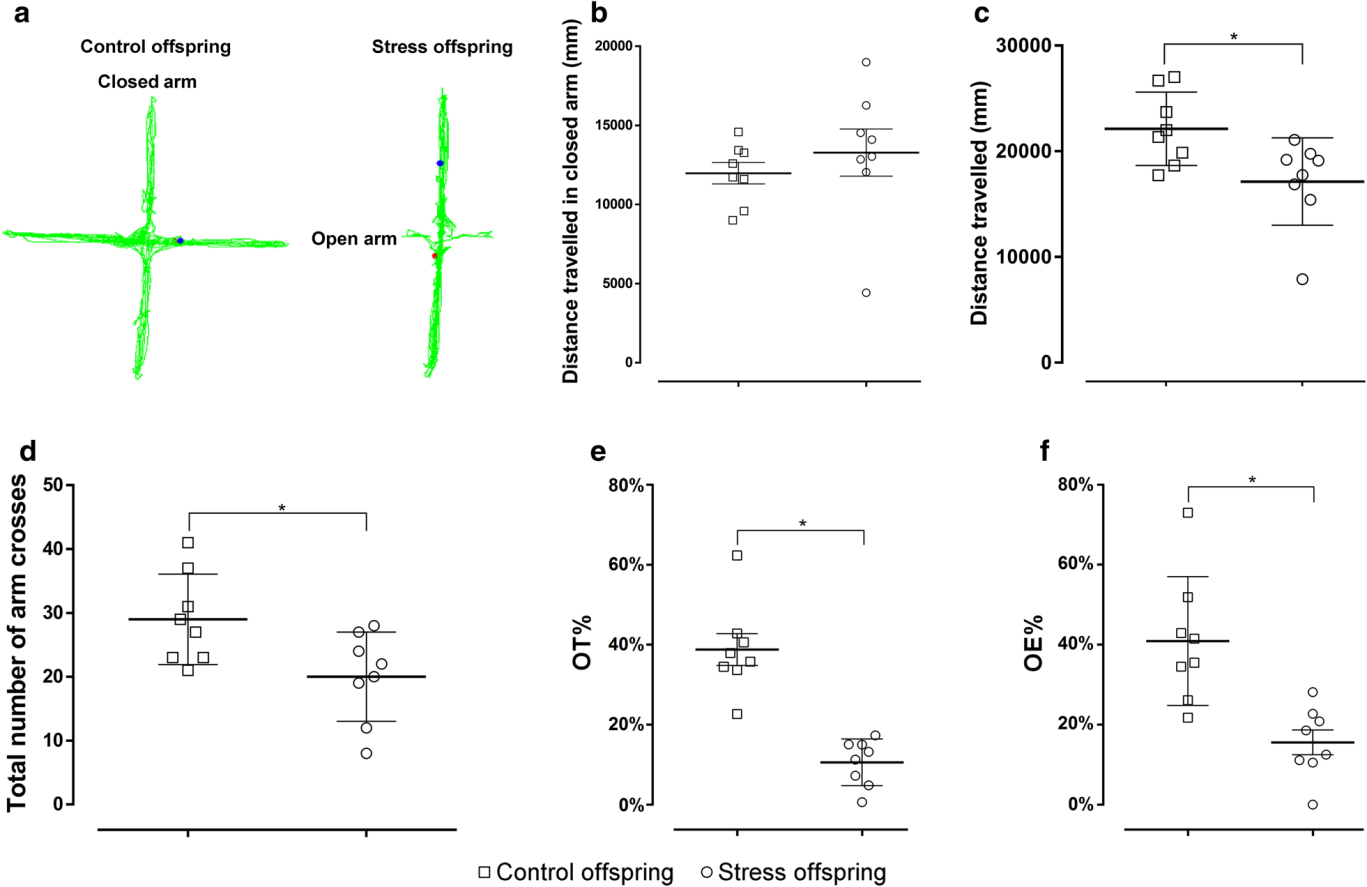
Elevated plus maze results for stress and control offspring. **a** Trajectory for both groups. **b** The distance travelled (mm) in the closed arms for both groups. **c** The total number of arm crosses for both groups. **d** The total distance (mm) travelled for both groups. **e** OT% [time spent in open arm (s)/total staying time (s)]. **f** OE% (number of entries into the open arms/total number of entries into the open arms, closed arms, and centre area). Control offspring: offspring of control group (hollow square, n = 8); stress offspring: offspring of maternal rats subject to SDS stimulation (hollow circle, n = 8); OT: open arm time; OE: open arm entry number; *p < 0.05

In the EPM, there was no significant difference in the distance travelled in the closed arm between the offspring subjected to pre-gestational SDS and control offspring (t(14) = 0.799, p = 0.4376, SDS offspring = 13,281 ± 1488 mm vs. control offspring = 11,975 ± 677.2 mm) (Fig. 7a, b). The total distance travelled in both the closed and open arms was 22,127 ± 1228 mm for the control offspring and 17,130 ± 1461 mm for the stressed offspring [t(14) = 2.617, p = 0.0203] (Fig. 7a, c). The total number of arm crosses was 29 ± 2.507 for the control offspring and 20 ± 2.471 for the stressed offspring [t(14) = 2.557, p = 0.0228] (Fig. 7d). Additionally, the offspring subjected to pre-gestational SDS showed a decrease in the percentage of open-arm staying time (OT%) and in the percentage of open-arm entries (OE %) compared to the control offspring (OT%: t(14) = 6.290, p < 0.05, SDS offspring = 10.56 ± 2.052% vs. control offspring = 38.74 ± 3.982%; OE%: t(14) = 3.906, p = 0.0016 [Student’s *t*-test], SDS offspring = 15.55 ± 3.113% vs. control offspring = 40.87 ± 5.685%; Fig. 7a, e, f).

**Fig. 8:**
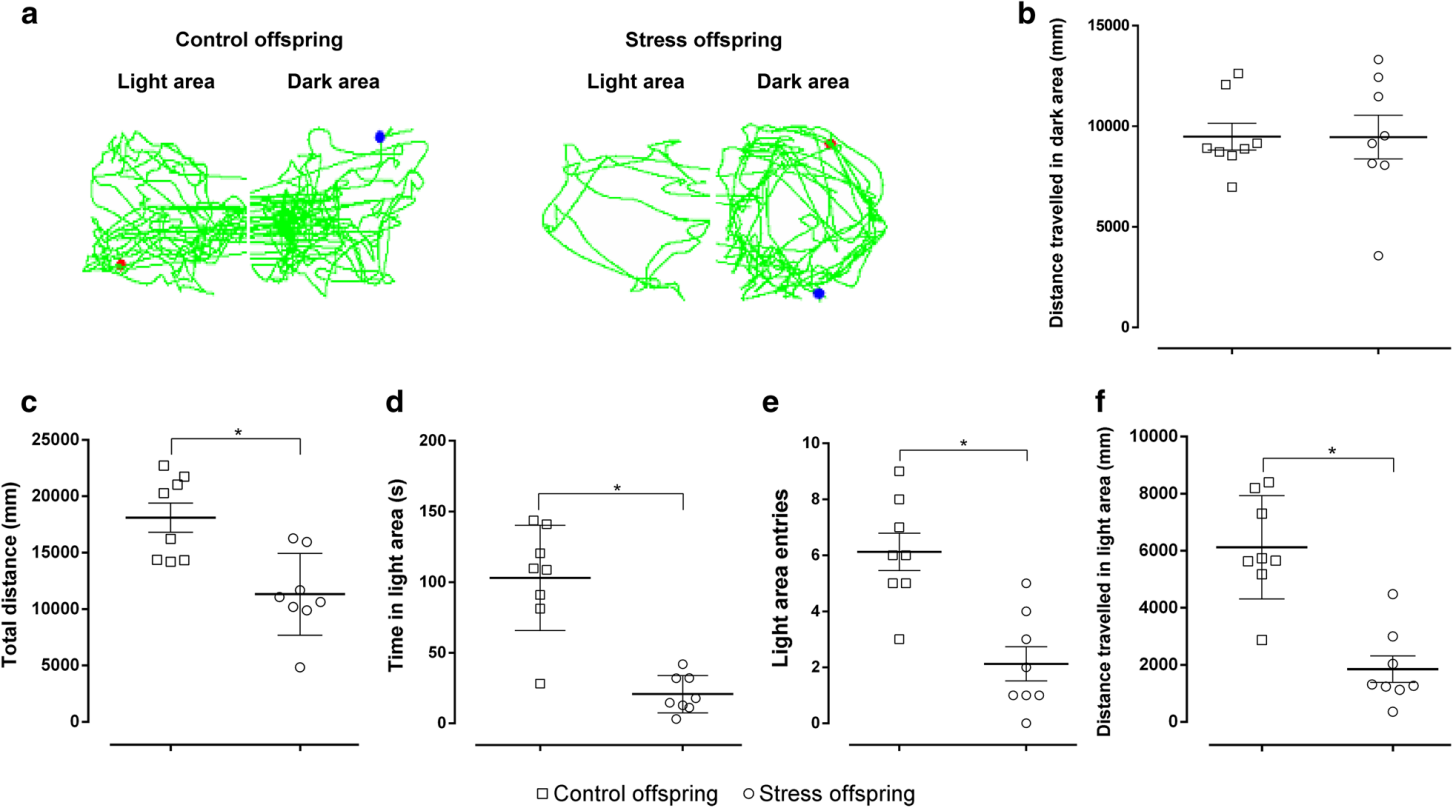
Light-dark box test results for stress and control offspring. **a** Trajectory for both groups. **b** The distance travelled (mm) in the dark area. **c** Total distance (mm) travelled in a 5 min period. **d** Time spent (s) in the illuminated area over a 5 min period. **d** Number of entries into the illuminated area. **e** Distance travelled (mm) in the illuminated area over a 5 min period. Control offspring: offspring of control group (hollow square, n = 8); stress offspring: offspring of maternal rats subject to SDS stimulation (hollow circle, n = 8); *p < 0.05

In the LDB, no difference was observed in the distance travelled in the dark compartment (t(14) = 0.01819, p = 0.9857, SDS offspring = 9461 ± 1088 mm vs. control offspring = 9485 ± 669.7 mm) (Fig. 8a, b). However, there was a remarkable reduction in the total distance travelled between the SDS offspring and controls (t(14) = 3.718, p = 0.0023 [Student’s *t*-test], SDS offspring = 11,316 ± 1282 mm vs. control offspring = 18,108 ± 1302 mm; Fig. 8a, c). The offspring subjected to pre-gestational SDS spent less time in the light, made less light-side entries, and moved less in the light side compared with the controls (time in light: t(14) = 5.895, p < 0.05, SDS offspring = 20.69 ± 4.662 s vs. control offspring = 103 ± 13.15 s; Light-side entries: t(14) = 4.425, p = 0.0006, SDS offspring = 2.125 ± 0.6105 vs. control offspring = 6.125 ± 0.6665; Distance travelled in light area: t(14) = 5.4, p < 0.05 [Student’s *t*-test], SDS offspring = 1855 ± 463.2 mm vs. control offspring = 6123 ± 640.6 mm; Fig. 8a, d–f).

**Fig. 9:**
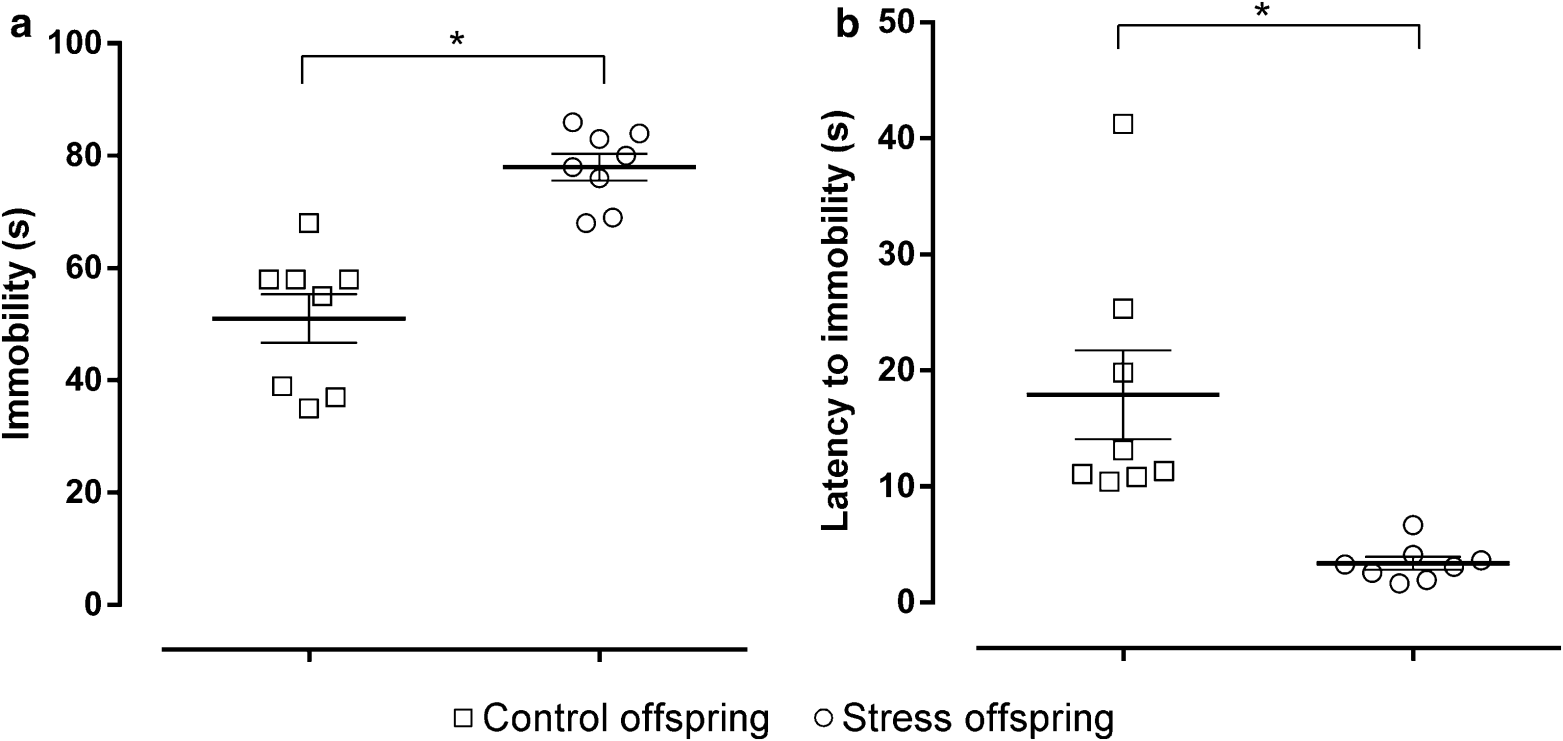
Forced swim test results for stress and control offspring. **a** Immobility time (s) over a 5 min period. **b** Latency to immobility [time to immobility (s)]. Control offspring: offspring of control group (hollow square, n = 8); Stress offspring: offspring of maternal rats subject to SDS stimulation (hollow circle, n = 8); *p < 0.05

In the FST, the offspring of SDS dams showed increased immobility and a decreased latency to immobility compared to the controls (immobility: t(14) = 5.475, p < 0.05, SDS offspring = 78 ± 2.368 s vs. control offspring = 51 ± 4.326 s; latency to immobility: t(14) = 3.752, p = 0.0021 [Student’s *t*-test], SDS offspring = 3.362 ± 0.5545 s vs. control offspring = 17.89 ± 3.834 s; Fig. 9a, b).

### Effect of offspring subjected to pre-gestational SDS on cognition

**Fig. 10:**
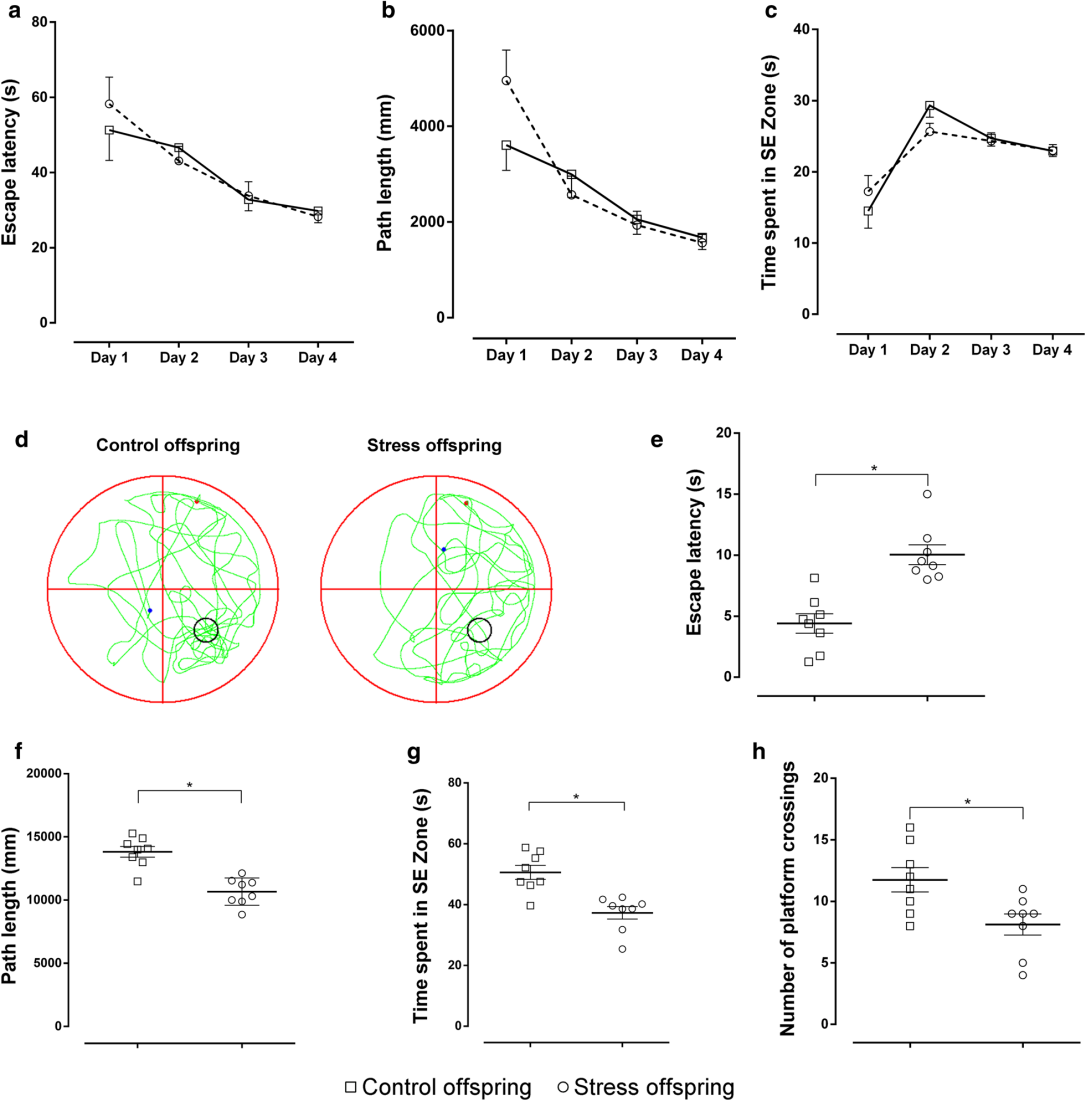
Morris water maze test for stress and control offspring. **a** Escape latency [total time taken to find the platform (s)] in the training trial. **b** Path length [distance swum to find the platform (mm)] in the training trial. **c** Time spent in the SE zone (s) in the training trial. **d** Trajectory for both groups in the water maze in the probe trial. **e** Escape latency (s) in the probe trial. **f** Path length (mm) in the probe trial. **g** Time spent (s) in the SE zone in the probe trial. **h** Number of times the platform was crossed in the probe trial. Control offspring: offspring of control group (hollow square, n = 8); Stress offspring: offspring of maternal rats subject to SDS stimulation (hollow circle, n = 8); SE zone: southeast zone; *p < 0.05

In the acquisition phase of the MWM task, the overall analysis (repeated measures ANOVA) revealed no significant effect of pre-gestational stress on escape latency, distance swum to find the platform, or time spent in the SE zone (Fig. 10a–c). However, pre-gestational stress induced a significant impairment in the retention phase, that is, the stressed offspring had a prolonged escape latency compared to controls (t(14) = 4.984, p = 0.0002 [Student’s *t*-test], SDS offspring = 10.03 ± 0.8083 s vs. control offspring = 4.394 ± 0.7917 s; Fig. 10d, e). The distance swum by animals searching for the platform, time spent in the SE zone, and platform crossings were significantly lower in the offspring of SDS dams compared to controls (distance swum: t(14) = 5.516, p < 0.05, SDS offspring = 10,668 ± 382.3 mm vs. control offspring = 13,819 ± 424.5 mm; time spent in the SE zone: t(14) = 4.32, p = 0.0007, SDS offspring = 37.28 ± 2.047 s vs. control offspring = 50.58 ± 2.301 s; Platform crossings: t(14) = 2.763, p = 0.0152 [Student’s *t*-test], SDS offspring = 8.125 ± 0.8543 vs. control offspring = 11.75 ± 0.9955; Fig. 10f–h).

**Fig. 11:**
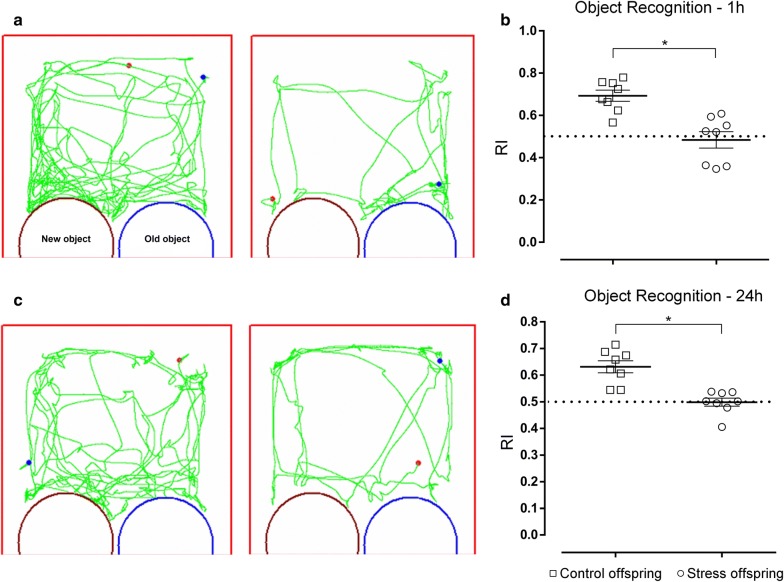
Object recognition task results for stress and control offspring. **a** Trajectory for both groups in the object recognition task with a 1 h retention. **b** Recognition index of the 1 h retention [time exploring unfamiliar object (s)/total exploration time (s)]. **c** Motion curves for both groups in the object recognition task with a 24 h retention. **d** Recognition index for the 24 h retention. Control offspring: offspring of control group (hollow square, n = 8); stress offspring: offspring of maternal rats subject to SDS stimulation (hollow circle, n = 8); RI: recognition index; *p < 0.05

In the novel object recognition test, offspring of stressed rats showed recognition memory deficits because the discrimination index was significantly lower for both the object recognition task with 1 h and 24 h memory retention (1 h retention: t(14) = 4.472, p = 0.0005, SDS offspring = 0.4844 ± 0.03877 vs. control offspring = 0.6935 ± 0.02613; 24 h retention: p = 0.0002 [Student’s *t*-test], SDS offspring = 0.4987 ± 0.01535 vs. control offspring = 0.6314 ± 0.02255, t(14) = 4.868; Fig. 11).

### HPA responsiveness in offspring

**Fig. 12:**
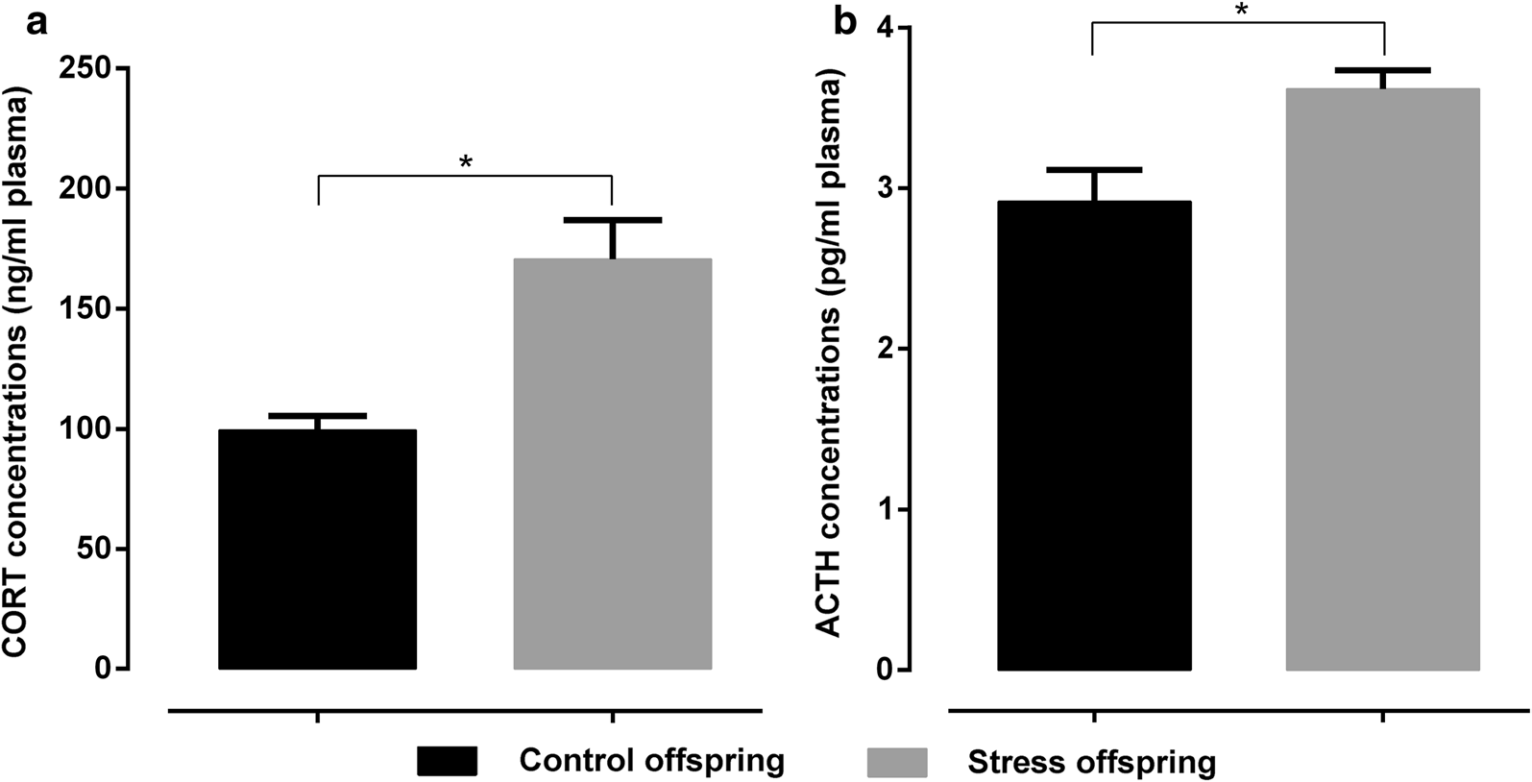
**a** CORT plasma concentrations (ng/ml plasma) in stress and control offspring (n = 6, outlier number = 2 for Control offspring; n = 7, outlier number = 1 for Stress offspring). **b** ACTH plasma concentrations (pg/ml plasma) in stress and control offspring (n = 8 for Control offspring; n = 8 for Stress offspring). Control offspring: offspring of control group (black); Stress offspring: offspring of maternal rats subject to SDS stimulation (grey); CORT: corticosterone; ACTH: adrenocorticotropic hormone; *p < 0.05

As shown in Fig. 12, corticosterone and ACTH levels in the pre-gestationally stressed offspring were significantly higher than those of the control offspring (corticosterone: t(11) = 3.875, p = 0.0026, ACTH: t(14) = 3.045, p = 0.0087 [Student’s *t*-test]). The corticosterone levels were 170.7 ± 16.14 ng/ml and 99.52 ± 5.892 ng/ml for pre-gestationally stressed and control offspring, respectively, while ACTH values were 3.619 ± 0.1173 pg/ml and 2.918 ± 0.198 pg/ml, respectively.

### Involvement of the monoaminergic system in the effects of pre-gestational stress in offspring

**Fig. 13:**
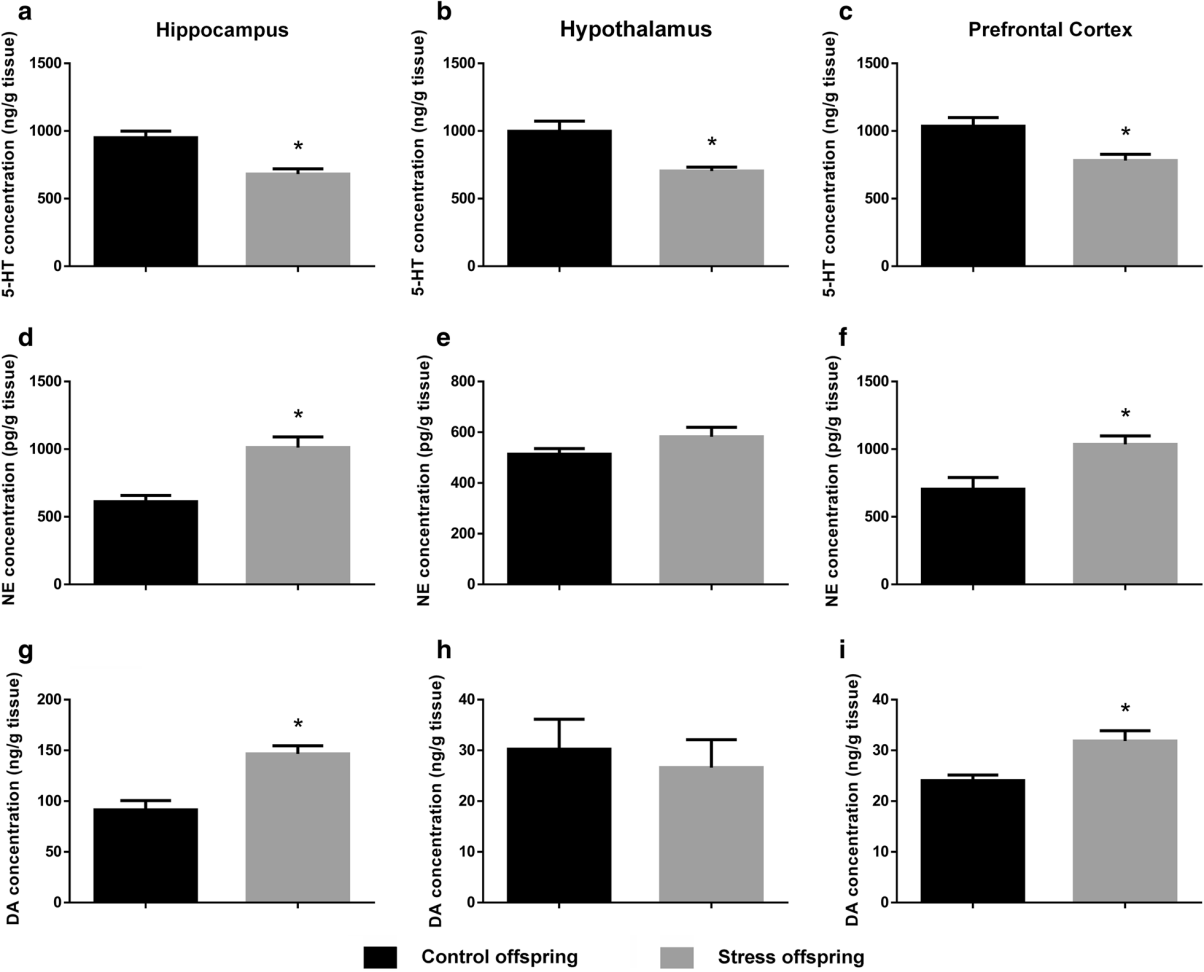
**a** 5-HT levels (ng/g tissue) in the hippocampus of stress (n = 8) and control offspring (n = 7, outlier number = 1). **b** 5-HT levels (ng/g tissue) in the hypothalamus of stress (n = 8) and control offspring (n = 7, outlier number = 1). **c** 5-HT levels (ng/g tissue) in the prefrontal cortex of stress (n = 7, outlier number = 1) and control offspring (n = 6, outlier number = 2). **d** NE levels (pg/g tissue) in the hippocampus of stress (n = 7, outlier number = 1) and control offspring (n = 8). **e** NE levels (pg/g tissue) in the hypothalamus of stress (n = 8) and control offspring (n = 8). **f** NE levels (pg/g tissue) in the prefrontal cortex of stress (n = 5, outlier number = 3) and control offspring (n = 6, outlier number = 2). **g** DA levels (ng/g tissue) in the hippocampus of stress (n = 6, outlier number = 2) and control offspring (n = 6, outlier number = 2). **h** DA levels (ng/g tissue) in the hypothalamus of stress (n = 6, outlier number = 2) and control offspring (n = 6, outlier number = 2). **i** DA levels (ng/g tissue) in the prefrontal cortex of stress (n = 5, outlier number = 3) and control offspring (n = 5, outlier number = 3). Control offspring: offspring of control group (black); Stress offspring: offspring of maternal rats subject to SDS stimulation (grey); 5-HT: serotonin; NE: norepinephrine; DA: dopamine; *p < 0.05

5-hydroxytryptamine levels were significantly lower in offspring of SDS dams compared to control offspring in the hippocampus (t(13) = 4.382, p = 0.0007, SDS offspring = 682 ± 38.57 ng/g vs. control offspring = 950.8 ± 48.64 ng/g), the hypothalamus (t(13) = 3.826, p = 0.0021, SDS offspring = 704.6 ± 28.38 ng/g vs. control offspring = 998 ± 75.64 ng/g), and the prefrontal cortex (t(11) = 3.273, p = 0.0074 [Student’s *t*-test], SDS offspring = 782.2 ± 46.36 ng/g vs. control offspring = 1035 ± 63.81 ng/g; Fig. 13a–c).

However, noradrenaline and dopamine levels were significantly higher in the offspring of pre-gestationally stressed rats compared to the offspring of control rats both in the hippocampus (noradrenaline: t(13) = 4.533, p = 0.0006, SDS offspring = 1012 ± 78.51 pg/g vs. control offspring = 611.4 ± 46.49 pg/g; dopamine: t(10) = 4.565, p = 0.001, SDS offspring = 146.6 ± 7.996 ng/g vs. control offspring = 91.45 ± 9.052 ng/g) and in the prefrontal cortex (noradrenaline: t(9) = 2.979, p = 0.0155, SDS offspring = 1036 ± 62.13 pg/g vs. control offspring = 703.8 ± 87.13 pg/g; dopamine: t(8) = 3.352, p = 0.01 [Student’s *t*-test], SDS offspring = 31.84 ± 2.055 ng/g vs. control offspring = 24.04 ± 1.091 ng/g; Fig. 13d, g, f, i).

### Effects of pre-gestational SDS on the expression of BDNF, pCREB, and SERT in the hippocampus and the prefrontal cortex of offspring

**Fig. 14:**
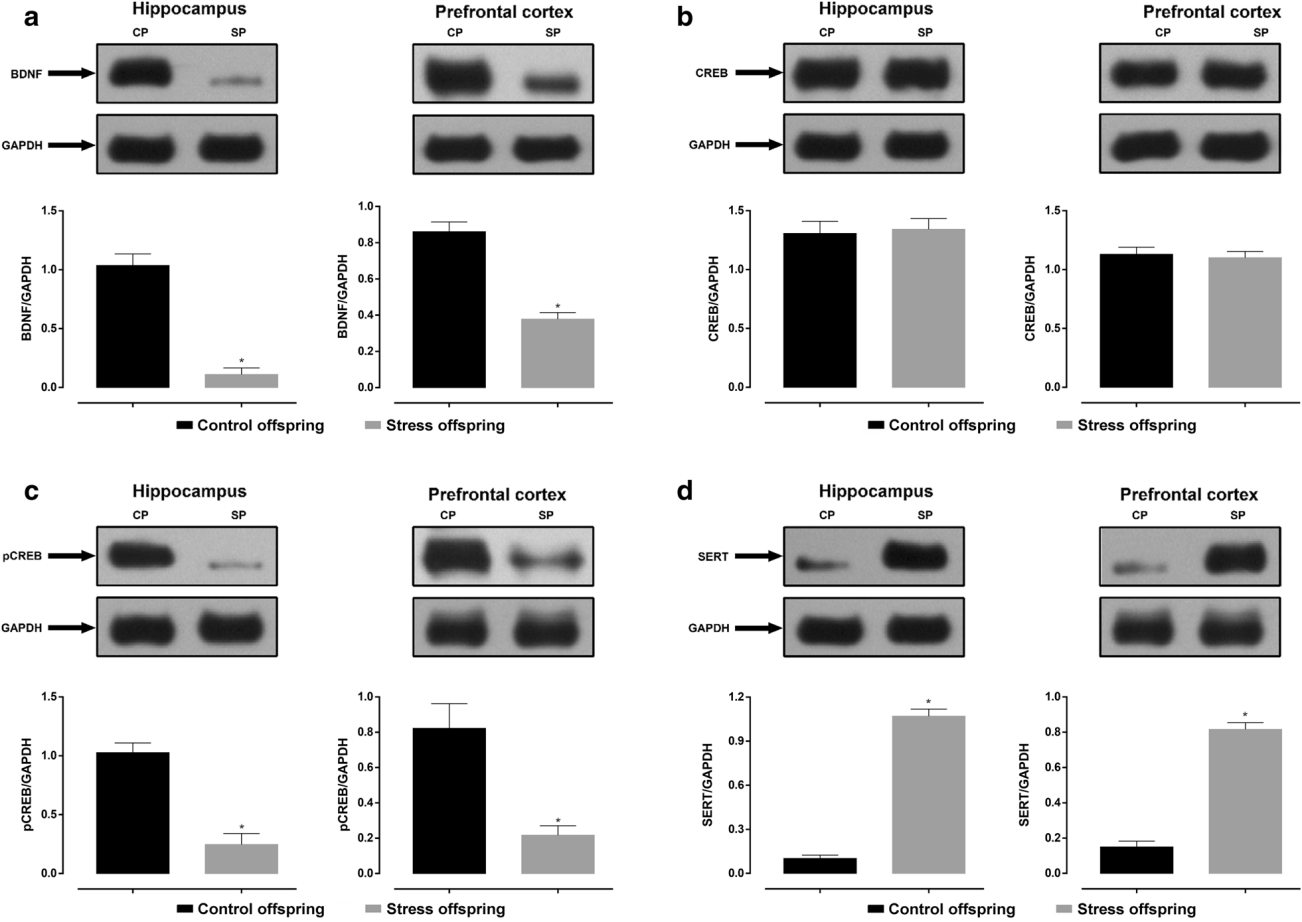
**a** Relative levels of BDNF/GAPDH in the hippocampus and the prefrontal cortex of stress and control offspring. **b** Relative levels of CREB/GAPDH in the hippocampus and the prefrontal cortex. **c** Relative levels of pCREB/GAPDH in the hippocampus and the prefrontal cortex. **d** Relative levels of SERT/GAPDH in the hippocampus and the prefrontal cortex. Control offspring (also CP): offspring of control group (black, n = 4); stress offspring (also SP): offspring of maternal rats subject to SDS stimulation (grey, n = 4); BDNF: brain derived neurotrophic factor; CREB: cAMP-response element binding protein; pCREB: phosphorylated cAMP-response element binding protein; SERT: serotonin transporter; GAPDH: glyceraldehyde-3-phosphate dehydrogenase; *p < 0.05

As shown in Fig. 14a, c, the relative optical density of BDNF and pCREB in the hippocampus and the prefrontal cortex of pre-gestationally stressed offspring was significantly lower in controls (BDNF hippocampus: t(6) = 8.228, p = 0.0002, SDS offspring = 0.1099 ± 0.05645 vs. control offspring = 1.037 ± 0.09748; BDNF prefrontal cortex: t(6) = 7.413, p = 0.0003, SDS offspring = 0.3782 ± 0.0363 vs. control offspring = 0.8606 ± 0.05401; pCREB hippocampus: t(6) = 6.239, p = 0.0008, SDS offspring = 0.2464 ± 0.09318 vs. control offspring = 1.026 ± 0.08334; pCREB prefrontal cortex: t(6) = 4.038, p = 0.0068 [Student’s *t*-test], SDS offspring = 0.217 ± 0.05386 vs. control offspring = 0.8223 ± 0.1399).

As shown in Fig. 14b, there was no significant difference between the relative optical density of CREB in the hippocampus and prefrontal cortex of pre-gestationally stressed offspring and controls (hippocampus: t(6) = 0.2529, p = 0.8088, SDS offspring = 1.342 ± 0.0916 vs. control offspring = 1.307 ± 0.103; prefrontal cortex: t(6) = 0.3728, p = 0.7221 [Student’s *t*-test], SDS offspring = 1.101 ± 0.05346 vs. control offspring = 1.13 ± 0.05984).

There was a significant increase in the relative optical density of SERT in the hippocampus and prefrontal cortex of SDS offspring compared to controls (hippocampus: t(6) = 17.95, p < 0.0001, SDS offspring = 1.07 ± 0.04871 vs. control offspring = 0.1014 ± 0.02325; prefrontal cortex: t(6) = 13.18, p < 0.0001 [Student’s *t*-test], SDS offspring = 0.8166 ± 0.03799 vs. control offspring = 0.1496 ± 0.03341; Fig. 14d).

## Discussion

### Chronic SDS in dams induces depressive-like behaviours

The first step in our investigation was to analyse whether the stress paradigm was effective or not in triggering the stress response in adult female rats. The dams exposed to social isolation and defeat stress showed lower body weight gain than the control group (Fig. 2), and this was comparable to results that have previously been reported in studies using a similar stress paradigm [40–42]. The limbic brain regions of the rats exposed to chronic stress were reported to show increased glutamate release [43], which in turn increased the demand for energy provided through lipid catabolism [44].

OFT was also designed to confirm the effect of our social defeat stress. Both the affective and motivational status of an animal can be determined by the initial activity of the animal when placed in an unfamiliar environment [29, 45], and it is generally believed that the responses of animals to an open but inescapable area are reflections of both the stress and reward induced by unfamiliar things [29]. In rats, reduced exploratory behaviour in an unfamiliar environment reflects a ‘refractory loss of interest’ [46] that is related to anhedonia [46, 47] because strange things are normally seen as rewarding in rats [47]. Exploratory behaviour, defined as the time taken by animals to explore unfamiliar environments, is very sensitive to past stress experiences [48]; however, the effects of physical stress tend to become attenuated following increased exposure to the stressors [49]. In the current paradigm, we explored the effects of chronic and sub-chronic social stress on open-field activities. Four weeks of social isolation and defeat stress significantly reduced the activity of stressed rats (Fig. 4b), and similar results have also been shown in a different SDS paradigm with adult rats and mice [29, 40, 42, 43]. Reduced exploratory behaviour in unfamiliar environments is also associated with enhanced anxiety levels in stressed animals [29], which is shown by reductions in the distance travelled in the central area and the time spent in the central area in stressed animals compared to controls (Fig. 4d, e). Similar results were also found [50] using Lewis rats exposed to SDS, in line with our findings. The unidirectional effects of social stress on locomotion (Fig. 4b, d, e) and rearing (Fig. 4f) suggest that the parameters chosen for exploratory activity should be dependent on the overall activity of the animals [29]. This is consistent with a report by Thiel et al. [51], who suggested that rearing and locomotor activity are related. It is noteworthy that there are also reports on the absence of any relationships between those two behaviours [52], and that is why we analysed all parameters of the OFT separately.

Over time, the rats exposed to chronic social stress showed decreased sucrose preference (Fig. 3), indicating a reduction in the sensitivity of stressed rats to reward, which is possibly homologous to anhedonia [53]. Similar effects have also been observed in other SDS paradigms [29, 40, 54].

Our data show that the chronic SDS experienced by rats induces depressive-like behavioural changes similar to those observed in humans [53, 55]. According to the DSM-IV criteria of the American Psychiatric Association, anhedonia occurs in patients with depression and opioid withdrawal and is characterised by a loss of interest and an incapacity to feel pleasure and joy, even in the case of normal positive stimuli. In our model, the reduced sucrose preference suggests desensitisation of the brain’s reward mechanism, while reduced locomotor activity and exploratory behaviours represent a lack of interest in unfamiliar stimuli, which may be indicative of motivational deficits. Anhedonia is one of the core symptoms of depression, and our results suggest that the current SDS paradigm might reliably simulate human depressive-like symptoms in rats.

### Chronic SDS in dams induces anxiety- and depressive-like behavioural changes accompanied by cognitive impairments in offspring

The male offspring of the dams exposed to SDS showed a profound decrease in the total path length travelled, path length travelled in the central area, time spent in the central area, and number of rearings in the open field compared with the offspring of the control group. This indicates that the offspring of stressed rats have significantly decreased locomotor and exploratory activities. These behavioural changes are consistent with those of stressed dams.

The offspring of the stressed dams showed a reduction in sucrose preference and relative sucrose preference, which was analysed to eliminate the effects of body weight on sucrose intake [5] (Fig. 6a, b). The decrease in sucrose intake represents a lack of preference for the sucrose solution [56], which is considered to be indicative of anhedonia [24]. Therefore, the results of our sucrose preference experiment show that the offspring of dams displaying depressive-like behaviours are at risk for developing depressive-like behaviours themselves.

The LDB and EPM are the most commonly used unconditioned tests to measure spontaneous approach-avoidance behaviours [57–59]. The time spent in the illuminated area, the number of entries into the illuminated area, and the distance travelled in the illuminated area in the LDB, as well as the OT% and OE% in the EPM, are mainly related to anxiety-like behaviours of animals [35, 60]. It has been shown that stressed male rats display significantly enhanced anxiety-like behaviours compared with controls and that SDS significantly decreased OT% in the EPM in rats [61]. On the other hand, the total path length travelled in the LDB and the EPM mainly reflects the overall activity of the animals [60], and we found that the offspring of stressed dams seemed to be significantly less active than the offspring of the control group, which is consistent with the results shown with regard to the total distance travelled in the OFT (Figs. 5, 7, 8). In order to overcome the problem of locomotor activity observed in the EPM and the LDB, we also measured the distance travelled in the closed arms of the EPM as well as in the dark compartment of the LDT, and found no difference in these measures between the groups (Figs. 7b, 8b). This suggests that stressed offspring indeed display an anxiety-like phenotype that is not due to a locomotor activity deficit. Thus, our results from the EPM and LDB indicate that the offspring of stressed dams show depressive and anxiety-like behaviours that were induced and occurred in their mothers.

The FST is the most widely used tool for the preclinical evaluation of antidepressant activity, and immobility in the FST is thought to reflect the failure of persistent escape-oriented behaviours and the development of passive behaviours from an active coping style towards stressful stimuli in animals. The reduction in immobility time after antidepressant treatment reflects the antidepressant activity of a drug [10]. In our experiment, the offspring of the stressed group showed increased immobility and a decreased latency to immobility compared to controls (Fig. 9). This can be explained by the hypothesis that the increase in immobility time induced by psychosocial stress is indicative of depressive-like behaviour [62, 63], which is representative of the loss of motivation and behavioural despair observed in patients with depression [10].

The spatial memory test showed that the memory acquisition ability of the offspring of the stressed group in the MWM was normal but that their ability to retrieve memories was impaired. As such, although there were no differences in escape latency, platform quadrant swimming distance, and the time spent swimming between the offspring of the stressed and control groups in the training phase (Fig. 10a–c), the benefits obtained from the previous long-term retention test were decreased. This manifested as a prolonged escape latency in the probe phase of the MWM and a significant decrease in swimming distance, time in the target quadrant, and number of platform crossings (Fig. 10f–h).

The ORT is used to assess recognition memory in animals, namely, non-spatial memories of facts and events [36]. In the ORT training phase, the offspring from the different treatment groups spent the same amount of time exploring the same two objects (RI > 50%), which indicates that exploration of unfamiliar objects takes longer than the exploration of familiar objects. However, after 1 h and 24 h memory retention, the offspring from the stressed group showed a significant decrease in the RI value, indicating a lower preference for unfamiliar objects compared to the offspring from the control group (Fig. 11).

In general, our data show that chronic SDS in dams can induce anxiety- and depressive-like behavioural changes accompanied by cognitive impairments in their offspring.

### Behavioural abnormalities in offspring of stressed dams are related to changes in the hypothalamic–pituitary–adrenal (HPA)-axis, the monoaminergic system, and transcriptional factors

In rats, reduced body weight gain and increased adrenal gland weight are reliable indicators of stress [10, 64]. It has been reported that rats following SDS and chronic unpredictable mild stress (CUMS) paradigms have increased adrenal gland weight [10, 65]. The increased adrenal gland weight in stressed animals may reflect hyperactivation of the HPA axis [10]. This is also shown by the significant increase in plasma concentrations of ACTH and CORT in the offspring of stressed rats compared to controls (Fig. 12). These results suggest that the offspring of dams exposed to SDS have an over-activated HPA axis and increased ACTH and CORT secretion.

The serotonergic system and the noradrenergic system play key roles in regulating the functional neural circuits of the brain and are involved in hippocampal-dependent memory [19, 20]. All brain regions involved in the stress response, such as the hippocampus, the hypothalamus, and the prefrontal cortex, represent the most relevant areas for depression [21, 22]. A decrease in levels of 5-HT was observed in the hypothalamus, the hippocampus, and the prefrontal cortex (Fig. 13a–c), but an increase in NE levels was observed only in the hippocampus and the prefrontal cortex (Fig. 13e, f) of the offspring of the stressed group compared with controls. Substantial evidence has shown that there are abnormalities in the NE and 5-HT neurotransmitter systems in depression and anxiety, and most of the evidence supports an underactivation of the 5-HT system and complex dysfunction of the NE system (most of the results indicate overactivation of this system) [4, 66].

An earlier study has shown that the DA system in the brain plays a key role in modulating the social behaviours of experimental animals [67]. Defeat promotes dopamine metabolism in the mesencephalic cortex and mesencephalic marginal zones in rats and mice [68, 69]. In addition, social defeat-related clues shown to rodents that have previously experienced defeat increase the release of DA in the mesencephalic cortex zone and meso-nucleus accumbens [69, 70]. Our experimental results also confirmed this change (Fig. 13g–i). In fact, activation of the DA system in the mesencephalic cortex region is a well-known stress response, and a number of studies have shown that the conditional increase in the metabolism and release of DA in the mesencephalic cortical zone and meso-nucleus accumbens is related to aversive experiences [71, 72]. The DA projection in the midbrain prefrontal cortex is selectively affected by a variety of mild stressors [73–75]. Some reports have suggested that the activation of dopaminergic neurons in the prefrontal cortex reflects emotionality and anticipatory fear. It has been argued that the release of dopamine in stress-related brain regions may be involved in the execution of cognitive activities aimed at eliminating or responding to stressors [74, 76], while another hypothesis attributes changes in dopamine to emotional arousal and the attempt to deal with the social stressor [69].

Notably, these abnormalities of the HPA-related monoaminergic system were observed in offspring of SDS dams, not the SDS dams themselves, in the present study. Our experiments show that the exposure of dams to chronic SDS alters the emotional behaviour, learning, and memory functions of their offspring. This is also accompanied by in vivo abnormalities in HPA-related hormones, the monoaminergic system, and transcriptional regulation factors (Fig. 14) such as CREB and BDNF. The above findings are consistent with the report by Zhang et al. [18], who also showed that the oral administration of CORT to rats unexposed to SDS increased SERT protein levels in the dorsal raphe nuclei, the hippocampus, the frontal cortex, and the amygdala. Furthermore, they also found that using antagonists of the glucocorticoid receptor, mifepristone and spironolactone (both alone and in combination), inhibited the increase in SERT protein levels in cerebral regions induced by SDS or orally administered CORT. Therefore, the increase in SERT protein levels in the dorsal raphe nuclei and forebrain limbic structures induced by SDS is primarily induced by CORT via the corticosteroid receptors [18]. This links changes in HPA axis-modulating hormones to changes in monoamine neurotransmitter levels in the offspring in this study. We speculate that chronic SDS causes an imbalance in the parental neuro-endocrine-immune network that affects the secretion of HPA axis regulatory hormones through the mother-placenta-foetus interface.

It has been suggested that in addition to serving as a neurotrophic factor during development BDNF regulates synaptic plasticity [77] and is involved in stress-induced hippocampal adaptation and the pathogenesis of depression [78]. This viewpoint is supported by the following facts: (a) exogenous BDNF exerts antidepressant activity [79, 80], and (b) antidepressant therapy inhibits the stress-induced reduction in BDNF mRNA and protein in the hippocampus [81]. Stress-induced increases in glucocorticoids are accompanied by structural changes in certain brain regions, neuronal damage, as well as decreased BDNF expression in the hippocampus, which can be blocked by chronic electroconvulsive shock and antidepressant treatments [82]. The monoaminergic signalling pathway mainly acts through G proteins leading to changes in adenylate cyclase activity [83] and increases in circulating cAMP causing the activation of protein kinase A (PKA), which is reportedly increased after chronic antidepressant treatment [84]. A key molecule related to the long-term protein expression changes observed following the activation of PKA and other signal transduction pathways is the constitutively expressed transcription factor CREB [85]. pCREB can regulate a variety of target genes involved in the pathophysiology of depressive disorders [24, 85]. In our experiment, the relative levels of pCREB/GAPDH in the hippocampus and prefrontal cortex of the male offspring of stressed dams were significantly lower than that of controls (Fig. 14c). This result is in keeping with the findings of Honghai et al. [4, 22]. The possible mechanism therein is that CREB acts as a third messenger and through its activated form, pCREB, acts on the promoters (the so-called fourth messengers) of target genes (e.g., BDNF, dynorphin, fos, and corticotropin releasing factor [CRF]) via its cognate gene regulatory element CRE (cAMP response element), thus causing/mediating subsequent physiological responses [85]. Taking previously reported works and our findings together, we also speculate that abnormal fluctuations in these neurosteroids induce a change in the levels of proteins such as SERT via the glucocorticoid receptor, thus affecting levels of neurotransmitters such as 5-HT, NE, and DA. These neurotransmitters are involved in the activation of CREB as a first messenger and influence the expression and regulation of multiple genes relevant to depressive- and anxiety-like behaviours, ultimately leading to abnormal behavioural phenotypes in the male offspring of SDS dams. In fact, our results from the association analysis between offspring behavioural phenotype and neurochemical indices also showed that the total distance travelled in the OFT, sucrose preference in the SPT, and the recognition index in the ORT were significantly positively correlated with BDNF and pCREB levels and were significantly negatively correlated with SERT.

### Summary and limitations of current study

In summary, our results show that (a) the maternal social defeat paradigm may reliably imitate human depressive-like symptoms; (b) the maternal rats experiencing SDS pass their anxiety- and depressive-like behaviours on to their offspring; and (c) the abnormal behaviours observed in the offspring may involve HPA axis regulatory hormones, the monoaminergic system, and changes in transcriptional regulation factors such CREB and BDNF. Moreover, there is evidence to suggest that dams exposed to SDS also experience the same physiological and biochemical changes, showing a homology of behavioural phenotypes and neurobiochemical profiles between the dams and their offspring. Recently, many studies have presented evidence that parental influences on offspring occur through changes in the epigenetic modification in germline stem cells [86–89]. We were not sure if the influence of maternal SDS before pregnancy on offspring behaviours and neurological damages occurs in a similar manner, and this needs to be further studied.

This work has some potential limitations. First, we did not examine maternal care behaviours (e.g. nursing, grooming, etc.), which could have also been affected by SDS. The SDS dams also did not cross-foster the pups. As a result, the “passing on” behavioural deficits could be a mixture of nature and nurture.

Interestingly, other groups found that offspring of the socially defeated paternal side also display anxiety- and depression-related behaviours [90]. Although we used separately fed paternal rats with sexual experience and statistically equal open-field and sucrose preference scores as the mating partners of SDS maternal rats, we failed to thoroughly eliminate the adverse effect of individual variability in paternal stress history.

Another concern might be that blood and brain tissues were collected after a series of behavioural experiments. We did not assess whether the neurobiological outcomes would have been influenced by the behavioural tests. In other words, the previous experiments, especially the FST experiment [91], could have affected the results of the subsequent experiments.

In addition, only male offspring were experimentally evaluated in the current study to avoid interference of hormones with the behaviour of the offspring. However, exclusion of females could be a significant point against this study’s clinical relevance because depression and anxiety are more prevalent in women than in men. Actually, we noticed that some subtypes of depression or anxiety were related to fluctuating hormones [92, 93]. Evaluating only male offspring could focus more on the “passing on” behavioural deficits caused by maternal chronic SDS.

## Conclusion

Our findings show that maternal SDS before pregnancy can affect behaviour and related neurobiological mechanisms in the offspring. This results in a tendency for emotional disorders/diseases in the offspring, and such a tendency extends to adult stage even though the maternal rats subject to SDS stimulation might recover as time passes. This extends our understanding of the influence of parental stress on offspring, both before and during pregnancy.

## Abbreviations

SPF: specific pathogen-free
SDS: social defeat stress
CUS: chronic unpredictable stress
MWM: Morris water maze
SPT: sucrose preference test
OFT: open field test
EPM: elevated plus maze
LDB: light–dark box
ORT: object recognition task
RI: recognition index
FST: forced swimming test
CV: coefficient of variation
ANOVA: analysis of variance
HPA: hypothalamic–pituitary–adrenal
ACTH: adrenocorticotropic hormone
CORT: corticosterone
BDNF: brain-derived neurotrophic factor
CREB: cyclic adenosine monophosphate response element binding protein
pCREB: phosphorylated CREB
SERT: serotonin transporter
PKA: protein kinase A
NE: norepinephrine
5-HT: serotonin
DA: dopamine
